# Investigating Past, Present, and Future Trends on Interface Between Marine and Medical Research and Development: A Bibliometric Review

**DOI:** 10.3390/md23010034

**Published:** 2025-01-10

**Authors:** Mehdi Zamani, Tetyana Melnychuk, Anton Eisenhauer, Ralph Gäbler, Carsten Schultz

**Affiliations:** 1 Kiel Institute for Responsible Innovation, Chair of Technology Management, Kiel University (Christian-Albrechts-Universität zu Kiel), 24118 Kiel, Germany; zamani@bwl.uni-kiel.de (M.Z.); melnychuk@bwl.uni-kiel.de (T.M.); 2 GEOMAR Helmholtz-Zentrum für Ozeanforschung Kiel, 24148 Kiel, Germany; aeisenhauer@geomar.de (A.E.); rgaebler@geomar.de (R.G.)

**Keywords:** bibliometric analysis, text mining, science mapping, marine research, medical research

## Abstract

The convergence of marine sciences and medical studies has the potential for substantial advances in healthcare. This study uses bibliometric and topic modeling studies to map the progression of research themes from 2000 to 2023, with an emphasis on the interdisciplinary subject of marine and medical sciences. Building on the global publication output at the interface between marine and medical sciences and using the Hierarchical Dirichlet Process, we discovered dominating research topics during three periods, emphasizing shifts in research focus and development trends. Our data show a significant rise in publication output, indicating a growing interest in using marine bioresources for medical applications. The paper identifies two main areas of active research, “natural product biochemistry” and “trace substance and genetics”, both with great therapeutic potential. We used social network analysis to map the collaborative networks and identify the prominent scholars and institutions driving this research and development progress. Our study indicates important paths for research policy and R&D management operating at the crossroads of healthcare innovation and marine sciences. It also underscores the significance of quantitative foresight methods and interdisciplinary teams in identifying and interpreting future scientific convergences and breakthroughs.

## 1. Introduction

Advancements in technology and interdisciplinary research are increasingly leading to significant breakthroughs at the intersection of diverse scientific fields. This may be especially relevant for the convergence between marine sciences and medical sciences [1]. Appropriate techniques are needed to integrate the disparate components of science and technology convergence [2]. These kinds of convergences serve as catalysts for the development of innovative solutions and promote interdisciplinary collaboration capable of addressing complex global concerns. As the COVID-19 study highlights, a balanced approach for researching across intersecting domains is crucial because of the difficulties in maintaining interdisciplinarity and managing topic displacement when attention is narrowed to one area [3]. By integrating in this way, these partnerships go beyond what is practical in their respective fields.

The marine environment, recognized for its numerous ranges of life forms, is a treasure trove of bioactive compounds. These marine-based substances have more and more turned out to be critical for groundbreaking scientific improvements [4,5,6,7,8]. For instance, the use of bioactive compounds from marine sources has become increasingly significant due to their medical properties in treating various diseases. Marine natural products (MNPs) may exhibit a range of beneficial pharmaceutical activities such as antibacterial, antiviral, neuroprotective, anticancer, and anti-inflammatory effects [9,10]. Moreover, marine algae are recognized as an underutilized resource among marine life forms. Recent research has proved the various biological and neuroprotective properties of marine algae, such as their antioxidant capabilities, anti-neuroinflammatory effects, cholinesterase inhibition, and the ability to prevent neuronal death. Therefore, marine algae hold significant promise for applications in pharmaceuticals, nutraceuticals, and functional foods [11]. Furthermore, marine sponges are renowned for hosting a variety of microbes and serve as a major reservoir of bioactive compounds such as enzymes [9,12,13]. Other marine organisms such as marine fungi [14,15,16], L-asparaginase [17,18], tunicates, bryozoans, molluscs [19] gorgonians [20], seaweed, and microorganisms [21] also produce substances potentially beneficial for treating human illnesses. CRISPR-based technologies, such as those explored in the context of marine microbial systems, have been employed to promote genetic therapies [22,23,24]. Furthermore, marine-derived enzymes, such as laccases, have shown therapeutic promise in the treatment of metabolic and inflammatory disorders [25,26,27]. In addition, the implementation of specific scientific techniques, which were initially created within marine research, provides opportunities for a stronger interaction between the medical and marine sciences. Isotopic tracing technologies, which were originally created to examine marine nutrient cycles, have been modified to improve the identification of disease alterations in human tissues. As a result, these marine-derived techniques are essential for improving our comprehension of marine biology and have the potential to revolutionize medical diagnostics by providing earlier and more accurate illness detection [28,29,30]. Interdisciplinary collaboration on marine natural products has led to the creation of ecologically friendly medications with long-term applications in healthcare [31,32]. These instances demonstrate the transformative power of combining marine and medical sciences, which not only advances healthcare but also promotes sustainability. The synergy between these disciplines serves as a model for tackling global health challenges while maintaining marine biodiversity, demonstrating the importance of fostering cross-disciplinary collaboration for reaching the full potential of these disciplines [33].

The BlueHealthTech research and development program in northern Germany exemplifies a growing global effort to harness the untapped potential at the intersection of marine sciences and medical applications. Through interdisciplinary collaboration, the program focuses on the pioneering research that bridges two critical domains: ocean sciences and human health [34]. Several projects of the BlueHealthTech consortia aim to develop cutting-edge diagnostic methods that leverage tools and techniques traditionally used in the marine sciences. For instance, isotope trace analyses, a method commonly applied in studying marine environments, are being adapted to address pressing medical challenges. These include investigating diseases of the musculoskeletal system, e.g., osteoporosis, a condition linked to calcium metabolism; oxidative stress, which is a precursor to various cancers; and neurological diseases, where early detection remains a critical unmet need. By translating marine diagnostic tools into medical applications, BlueHealthTech is advancing early intervention strategies that could revolutionize healthcare. Another key research area focuses on the potential of micro- and macroalgae in contributing to human health. These marine organisms are rich in bioactive compounds that show promise for addressing a wide range of health challenges. BlueHealthTech projects are exploring applications such as the following: disease prevention through algae-derived antioxidants and anti-inflammatory compounds; cancer treatment, which targets specific pathways influenced by bioactive marine molecules; age-related macular degeneration, where algae-based therapies may help preserve vision; regulation of inflammation, which is a central factor in many chronic diseases. These efforts reflect the growing recognition of marine biodiversity as a rich source of untapped therapeutic potential. The program’s success is based on its diverse and collaborative ecosystem, which brings together stakeholders from various sectors, including the following: basic research institutions conducting foundational studies in marine and medical sciences; medical applications that translate scientific insights into clinical solutions; pharmaceutical and medical technology companies driving innovation and commercialization; and marine farms, which provide sustainable sources of marine-derived raw materials. This cross-sectoral collaboration ensures a seamless flow of knowledge, resources, and expertise, accelerating the transition from scientific discovery to real-world application. BlueHealthTech’s ambitious initiatives are made possible through public funding, which has currently secured over two three-year funding periods. This sustained support underscores the importance of investing in interdisciplinary R&D for addressing societal health challenges. The program has also attracted the attention of seed investors, who increasingly recognize the potential to transform promising innovations into valuable business cases. This alignment of public and private sector interest not only supports long-term sustainability but also catalyzes the creation of new market opportunities, especially in areas such as algae-based therapeutics and advanced diagnostics. The work conducted by BlueHealthTech has implications far beyond northern Germany. By fostering innovative research and translating marine science into medical advancements, the program contributes to global health and may serve as a model for interdisciplinary collaboration.

In this context, our primary intention in this study is to become aware of the key topics on the interface between marine and medical research and development, as well as to observe the changes within this intersection over time. We aim to recognize the tendencies and the development of studies at this nexus from the years 2000 to 2023. This examination is segmented into three periods of equal duration, 2000–2007, 2008–2015, and 2016–2023, through which an in-depth evaluation of the evolving dynamics in the convergent fields of marine and medical research is performed. We use topic modeling and bibliometric analysis to become aware of research traits, styles, and new topics, which examines the recent history of research and provides a thorough overview of the past, present, and capacity of this interdisciplinary field [35,36]. These kinds of methods have proven effective in identifying emerging themes, mapping technological convergence, and pinpointing key research trends, which can also be applied to exploring the evolving landscape at the convergence of marine and medical R&D [37,38,39,40]. We also identify the main contributors in this field, including individuals, institutions, and countries. This is accomplished by way of co-country, co-organization, and co-authorship analysis.

The findings shed light on the scientific dynamics within the interface between marine and medical research and development. These insights have implications for policymakers and R&D managers. They could help them allocate resources and develop strategic plans for medical advancement in this specific field of convergence. This encompasses potential innovation fields for drug discoveries from marine organisms, with marine-derived components being used in health prevention and clinical treatments, as well as the impact of marine biotechnology and scientific methods on medical diagnoses. This study is organized as follows: Section 2 describes the methodologies used in the bibliometric analysis, including the data mining method, topic extraction, social network analysis, and our study design. Section 3 shows the results, analysis of the findings, and discussion. Section 4 presents implications for research policy and management, Section 5 discusses limitations and directions for future research, and Section 6 presents concluding remarks on this work.

## 2. Methodology

**Figure 1:**
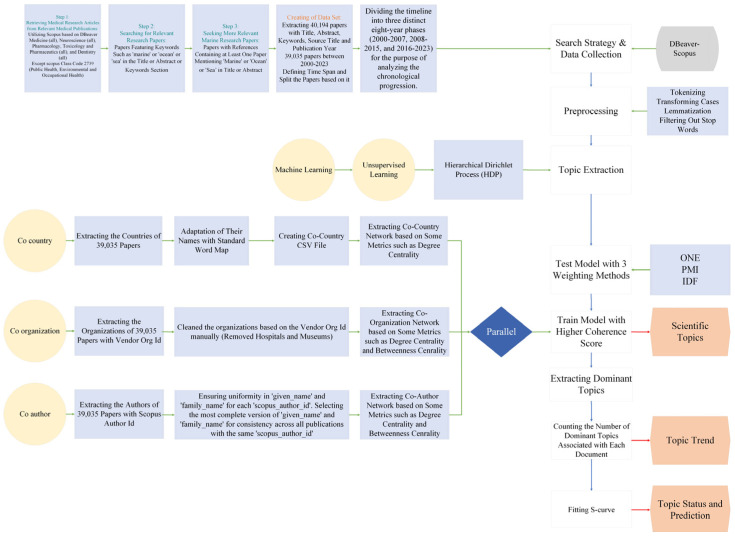
Research stages.

**Figure 2:**
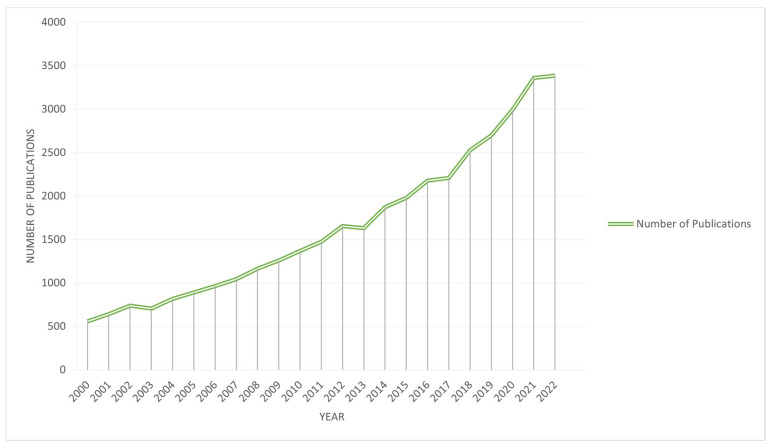
Number of publications per year.

We exploit the scientific literature of the entire period since the year 2000. The literature search was carried out by using the Scopus database. Scopus is a comprehensive bibliographic database that is comparable to or surpassing the coverage of articles by the Web of Science database [41,42]. It offers some advantages over alternative databases such as Web of Knowledge. This advantage comes in the form of the Scopus author ID, which makes it possible to identify and disambiguate unique authors accurately. This problem is critical for co-authorship and cooperation network analysis since it ensures that individual researchers’ contributions and affiliations are identified across the dataset. Furthermore, Scopus provides more international journal coverage and improved bibliometrics tools, allowing for large-scale data extraction and analysis. While the WoS gives useful information, Scopus has unique capabilities and more completely indexes itself within the context of this study. The analysis includes all available literature in the fields of Medicine, Neuroscience, Pharmacology, Toxicology, Pharmaceutics, and Dentistry. An exception was made to exclude documents categorized under the Scopus Class Code 2739, which relates to Public Health, Environmental, and Occupational Health. These documents were omitted because they frequently contain papers that are not directly relevant to the intersection of marine and medical sciences. This analysis excludes research that focuses solely on environmental health policies or occupational safety in non-marine environments. This exclusion allowed us to keep a focused dataset, preferring works that overlapped marine and medical disciplines. Subsequently, the data corpus was refined to focus on relevant marine-related research papers that especially include key phrases along with “marine”, “ocean”, or “sea” inside the title, abstract, or keywords section. In addition, we complement the breadth of relevant literature by including papers that covered references to at least one paper citing “marine”, “ocean”, or “sea” in the title or abstract. This step allowed us to identify marine studies papers in the overall corpus of medical research that might not have been captured throughout the initial keyword-primarily based search. (Step 1, Step 2, and Step 3 in Figure 1) This search strategy resulted in a data corpus of a total of 40.194 papers. Each paper within this dataset was extracted with its complete bibliometric information, including the title, abstract, keywords, etc. After limiting the time frame of the papers to the year 2000 to 2023, the number of papers was reduced to 39.035. The dataset was then organized by defining three periods of eight years (2000–2007, 2008–2015, and 2016–2023) to allow a longitudinal evaluation. The consistent annual increasing trend in the number of publications led to the division into three periods. Figure 2 shows that over the study period, the output of publications did not significantly fluctuate. Therefore, we divided the timeframe into three equal eight-year parts to assess long-term trends and shifts in research concentration throughout time.

The collected data underwent preprocessing, an important step to clean and prepare the data for analysis. This step included text manipulation along with tokenizing, case transformation, lemmatization, and stop word removal. During this step, raw textual content streams were first tokenized, changing sentences, terms, and words into processing gadgets called tokens without punctuation, including commas, colons, and spaces. Next, we standardized all characters in the corpus to the equal case (either lower or upper case). By lemmatization, we grouped specific inflected sorts of phrases collectively to be analyzed as a single object. Finally, filtering out stop words eliminated not unusual, insignificant words from the textual content, streamlining the dataset for the extraction of significant content material [43,44,45].

Following this, we carried out an unsupervised learning strategy called the Hierarchical Dirichlet Process (HDP) to facilitate topic extraction. The HDP model was trained using the following parameters: min_cf = 100, which filters out terms that occur in less than 100 documents in order to reduce noise and concentrate on significant patterns, while rm_top = 0 means that all most frequent terms are kept with any intentional loss of information for evaluation on weighted approaches such as IDF. The hyperparameters gamma = 0.001 and alpha = 0.001 put some limit on the number of key topics that can be relevant to specific documents while also limiting problems of sparse topic allocations across documents. To ensure reproducibility, the model is initialized with respect to randomization by specifying the seed (seed = 99,999). The training phase is concluded after 100 cycles to obtain consistent and stable themes. HDP is a nonparametric Bayesian method for clustering grouped information. This advanced technique applied a Dirichlet technique for every subgroup of statistics, taking into consideration the distribution of clusters [46]. The HDP supplied numerous advantages over the traditional Latent Dirichlet Allocation (LDA), along with automatic topic number determination, which removed the need for predefined numbers of topics, a hierarchical topic structure, which allowed for multilevel analysis of topic relationships, and topic sparsity, ensuring that the most applicable subjects had been recognized with excessive precision [47]. To ensure the coherence of the topics, a test model with three weighting methods was conducted. This involved considering every term equally by means of default, using Inverse Document Frequency (IDF) to reduce the weight of terms that occur too regularly throughout the data [48], and employing Pointwise Mutual Information (PMI) to highlight terms that have a high degree of association in the context of a specific document or the entire corpus [49]. Additionally, we applied the ONE metric that considers every term equal inside the corpus [50]. The final model was built by choosing the term weighting technique that produced the best coherence rating, resulting in the extraction of dominant topics. A dominant topic is identified based on the topic number that has the greatest percentage contribution in each document [51]. These subjects were visualized using word clouds and further analyzed to decide subject matter tendencies. The last stage of our analysis of relevant research topics was to look ahead in time by investigating the dynamics of topics based on the technology S-curve model. The life cycle models have been used in different management fields (strategy, marketing, and production) in order to represent the evolution of industries, products, technologies, etc. The most widespread among them, initially formulated by Levitt (1965) [52], is the S-curve that describes the different stages in the temporal evolution of the performance of a technology. In this study, the S-Curve model was employed to analyze and predict research trends over time. This model identifies growth stages, maturity, and potential saturation points of research themes, offering insights into their lifecycle and long-term potential.

In parallel, we applied social network analysis (SNA) [53], integrated with Python, to research co-country, co-organization, and co-authorship networks. Using criteria such as degree centrality and betweenness centrality [54], we supplied insights into the collaborative networks of the marine medical field of convergence and identified the most influential actors. Our network study initially concentrated on the co-country partnerships. First, we extracted country information from 39,035 publications. To resolve differences in nation naming practices across various databases, the countries’ names were then standardized using a word map, guaranteeing data consistency. We use co-country data to identify the relationships between the countries that indicate cooperative actions. We use these data to build a co-country network that includes measures like betweenness centrality and degree centrality. Then, we utilized an organized method to examine co-organizational relationships. Using each of the authors of the papers and their Scopus vendor organization ID, we were able to extract 18,097 organizations from the collection of 39,035 articles. After that, the data were carefully cleaned to eliminate non-research entities like general (not researching) hospitals and museums, making sure the network only included partnerships for pure research collaborations. Lastly, we used SNA measures to reveal the co-organization network, emphasizing the key institutions and their functions in fostering connections across various research groups. Finally, we used the Scopus author ID to identify the individual authors. We checked potential differences in names linked to each ID in order to provide consistency in author identification during the co-authorship network. For the publication history of each author to be accurately represented, this step was essential. Next, we determined which version of each author’s name was the most comprehensive and consistent across all publications with the same Scopus author ID. We created a co-author network of individual authors and applied established SNA metrics. As a result, we were able to recognize significant authors in the field of marine medical research and comprehend the cooperation structure.

The overall empirical study design is summarized in Figure 1.

## 3. Results and Discussion

### 3.1. Descriptive Analysis

Figure 2 shows the number of publications inside the converging area of marine and medical research and development per year, from 2000 to 2022. The graph does not include the number of publications for the year 2023. This is because our data collection was completed only up to April 2023. The graph shows a steady increase in the number of research papers. After a small publication volume in 2000, an upward trend occurred around 2003. From there, the number of publications grows substantially, with the highest point visible in 2022, which reached close to 3.500 publications and was seven times more than at the beginning. This increase could be because of greater funding and/or a growing focus on the significance of this field of study. From 2000 to 2007, the first observed period contained 6358 papers, laying the groundwork for future studies. Following this, production almost doubled between 2008 and 2015, with 12,406 papers published. Also, the latter period is characterized by a significant increase in the number of publications from 2016 to 2023, rising by 20,271.

We also looked at how research at the interface of marine and medical sciences evolved throughout the three time periods, from 2000 to 2023. We retrieved and downloaded the full list of Web of Science (WoS) journals and their categories. This enabled reliable journal categorization and analysis. The categories of each journal were retrieved and calculated for each period to determine the prevalence and trends in study topics. This method allowed for a more in-depth study of the changing research landscape, revealing prevalent themes and growing areas of research at the intersection of marine and medical sciences. (Appendix A). Table 1 demonstrates how, within the convergence of the marine and medical sciences, research goals and dominant fields have changed throughout time.

“Pharmacology and Pharmacy” has been the top category for all three periods, demonstrating a stable presence in research focus even though its share of all articles has decreased slightly from 7.7% to 6.6%. Strong stances in the fields of “Clinical Neurology” and “Neurosciences” reflect continued interest in these fields. Although it was stronger in the second period, “Medicine, General and Internal” surpassed neurosciences in the most recent period. Studies in “Immunology and Oncology” have continued to be important, especially Oncology, which has seen a notable increase lately. This rise in the study of cancer within the convergence of marine and medical fields is gaining momentum. The field of “Public, Environmental, and Occupational Health” has experienced growth, moving up from ninth to third place recently. This shift indicates a greater focus on health matters concerning the environment. “Microbiology” has also risen in importance, moving from a less prominent role to a more substantial one. This increase might be attributed to escalating concerns about microbial resistance and the urgent need for novel antibiotics. “Toxicology” and “Endocrinology and Metabolism” both have declining relative publication volumes but are still in the top 15, suggesting a continued but diminished focus.

The changes according to the relevance of research over the three time periods demonstrate how research at the convergence zone of marine and medical sciences is constantly evolving. There is a clear movement toward more comprehensive and practical research, particularly in fields that have a direct influence on public health and the treatment of diseases. In sum, the table shows that interdisciplinary themes are becoming more prevalent, particularly at the intersections of experimental, clinical, and environmental medicine. The growth of fields that transcend conventional bounds, such as microbiology, experimental medicine, and public health, is indicative of this.

### 3.2. Topic Extraction (2000–2007)

**Figure 3:**
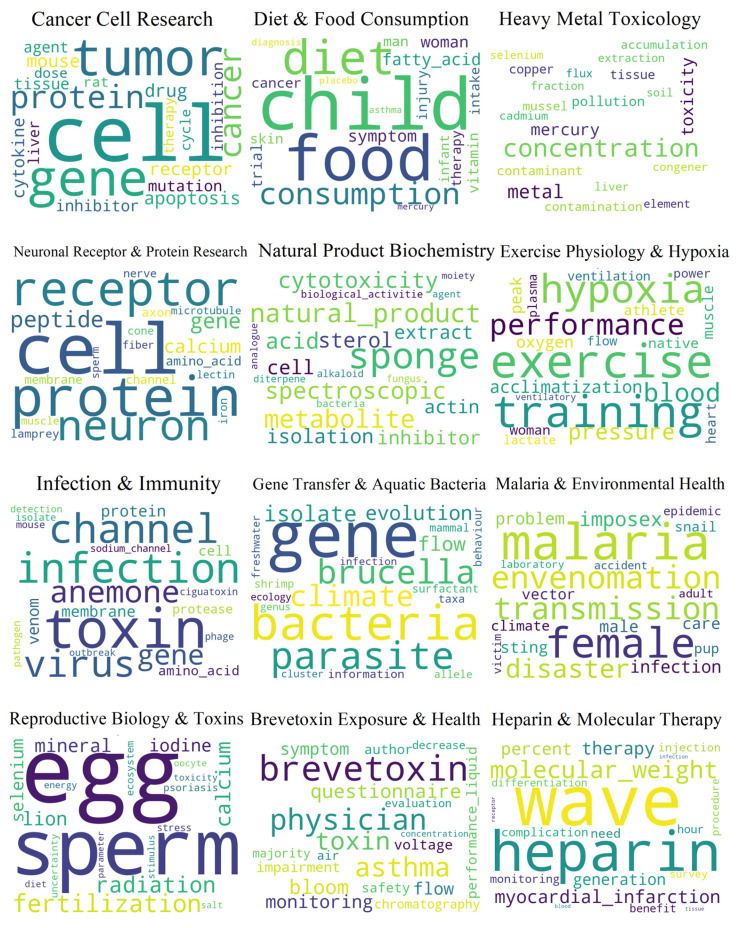
Hierarchical Dirichlet Process model 2000–2007.

Figure 3 delineates the topics from the Hierarchical Dirichlet Process (HDP) model applied to publications between 2000 and 2007 with its coherence score of the mentioned three weighting methods. This model facilitates the extraction of latent topics within the corpus, revealing the thematic foci of research at the interface between marine and medical research and development. Regarding the coherence scores, the IDF model outperforms other algorithms, achieving the highest coherence score (0.54) and thereby providing the most discernible and interpretable topics.

Accompanying the coherence score visualization, the detailed word clouds represent 12 distinct topics, each with a designated thematic title and description. It is important to note that the title of each topic in every period is derived from the most frequent keywords identified within that topic.

Firstly, “Infection and Immunity” likely encompasses studies on cellular infection, protein channels, and immune response to toxins and pathogens. Subsequently, “Exercise Physiology and Hypoxia” reflects research on the effects of exercise and oxygen deprivation on the body. Here, two different areas of oxygen-related research could be taken into consideration. The first one is Oxygen Consumption (VO2), which denotes the maximum oxygen intake a person is capable of achieving during vigorous or maximal exercise (VO2 max). This is a standard exercise physiology metric used to evaluate the aerobic capacity of athletes [55]. The second one, Blood Oxygen Saturation (SpO2), may examine how exercise and oxygen deprivation affect blood oxygen levels [56]. “Neuronal Receptor and Protein Research” encompasses studies on neuron receptors and related proteins. Furthermore, “Gene Transfer and Aquatic Bacteria” studies genetic flow in aquatic environments, focusing on bacteria like Brucella. In addition, “Heavy Metal Toxicology” reflects research on the accumulation and effects of heavy metals like selenium and mercury in biological systems. The description of accumulation generally involves the examination of tissue samples and biological monitoring. Tissue analysis concentrates on the substance within a specific organ that is recognized for its function in the detoxification and bioaccumulation of pollutants. Biological monitoring employs organisms like mussels, which are commonly used as sentinel species due to their ability to accumulate heavy metals in their tissues, thus indicating the level of contamination in their environment [57]. Following this, “Cancer Cell Research” focuses on the study of tumor cells, genes related to cancer, and cellular apoptosis. Moreover, “Natural Product Biochemistry” encompasses studies on bioactive compounds from natural sources like sponges. Correspondingly, “Diet and Food Consumption” reflects research on dietary habits, food intake, and their health implications. Continuing the thematic exploration, “Malaria and Environmental Health” focuses on the study of malaria, its transmission, and the influence of environmental factors. Additionally, “Reproductive Biology and Toxins” studies egg and sperm biology, including the impact of radiation and toxins on fertilization. Subsequently, “Heparin and Molecular Therapy” reflects research on heparin and its role in molecular therapies and medical treatments. Lastly, “Brevetoxin Exposure and Health”, focuses on the health effects of exposure to brevetoxins and related toxins.

During this period, there was a growing interest in the use of substances derived from the sea in medical applications, with a particular focus on discovering antimicrobial and immune-modulating agents. The therapeutic effects of bioactive compounds were of significant interest, especially in relation to cancer treatment and cardiovascular benefits from natural marine products. Diagnostic methods tended to focus on non-invasive monitoring, such as blood oxygen saturation during exercise, indicating a trend toward incorporating marine science techniques into clinical settings.

### 3.3. Topic Extraction (2008–2015)

**Figure 4:**
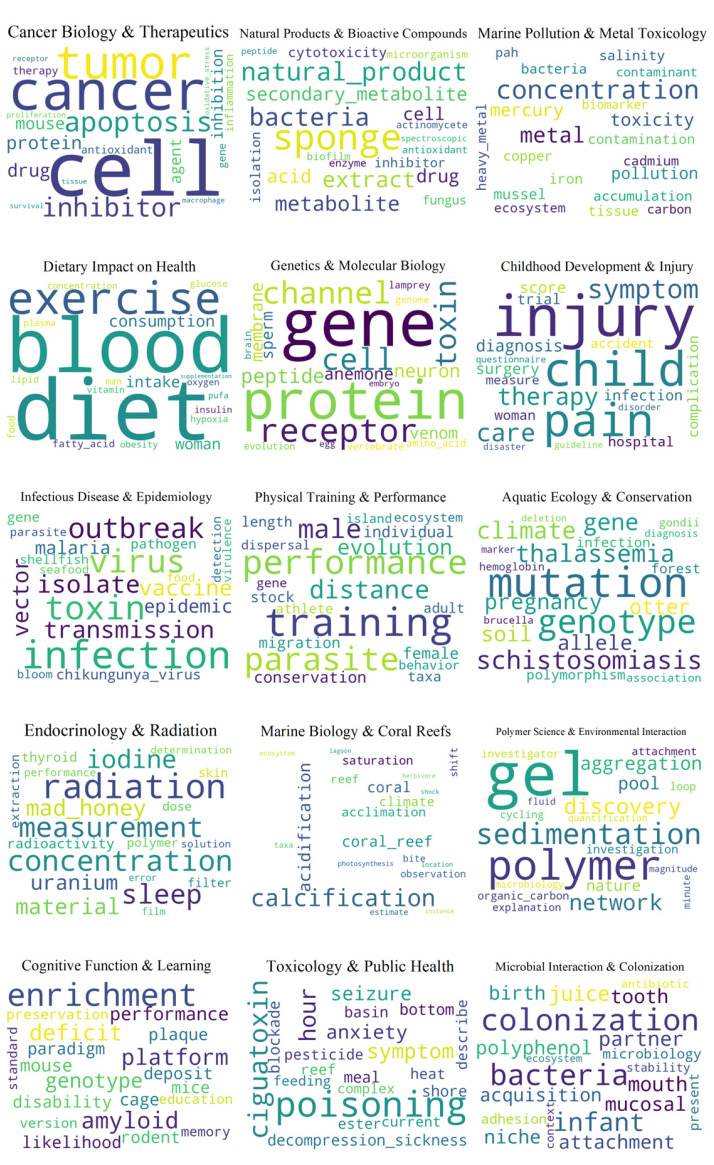
Hierarchical Dirichlet Process model 2008–2015.

Figure 4 shows the results of applying the Hierarchical Dirichlet Process (HDP) to research from 2008 to 2015. The coherence scores indicate the IDF model as the most effective, with a high score of 0.63, suggesting clearer, more interpretable topics. Additionally, word clouds depict 15 distinct topics, each with its title and description.

Initially, “Physical Training and Performance” likely deals with research into exercise, training, and their effects on the body, considering factors such as hypoxia. Then, “Aquatic Ecology and Conservation” covers studies on marine ecosystems, focusing on conservation, biodiversity, and the behavior of different species. “Infectious Disease and Epidemiology” encompasses research into pathogens, vectors, and the dynamics of disease transmission and outbreaks. Next, “Dietary Impact on Health” focuses on the effects of diet and nutrition on health, possibly including studies on obesity and metabolic factors. “Cancer Biology and Therapeutics” centers on cancer research, including tumor biology, gene expression, and drug development. Proceeding to the next topic, “Endocrinology and Radiation” investigates the impact of radiation on endocrine systems, with a focus on iodine and thyroid health. “Natural Products and Bioactive Compounds” studies the extraction and use of bioactive compounds from natural sources like sponges. Simultaneously, “Genetics and Molecular Biology” Involves research in genetics, gene expression, and the study of proteins and receptors. “Marine Pollution and Metal Toxicology” concerns environmental pollution, particularly with heavy metals, and their effects on marine and human life. “Childhood Development and Injury” addresses aspects of child health, including injury, therapy, and care. Advancing to the next topic, “Polymer Science and Environmental Interaction” involves the study of polymers and their interactions with the environment or within biological systems. Concurrently, “Cognitive Function and Learning” focuses on cognitive reserve, learning processes, and possibly the impact of toxins like brevetoxin on cognitive health. Then, “Marine Biology and Coral Reefs” encompasses studies on coral reefs, including aspects of calcification and photosynthesis in marine ecosystems. “Toxicology and Public Health” covers the study of various toxins, their sources, detection, and their health implications for humans. Finally, “Microbial Interaction and Colonization” investigates the colonization by bacteria and other microbes in various environments, including the oral cavity.

Research on the effects of marine life on human health grew between 2008 and 2015. These topics included the possible therapeutic use of naturally occurring marine substances to treat cancer and the ecological implications of marine conservation on human health. Further, understanding the long-term health effects of dietary practices influenced by marine food sources was a major area of focus. Improvements in measuring physiological responses to different health interventions indicate a clear push toward better and early detection and monitoring methods in terms of diagnostics.

### 3.4. Topic Extraction (2016–2023)

**Figure 5:**
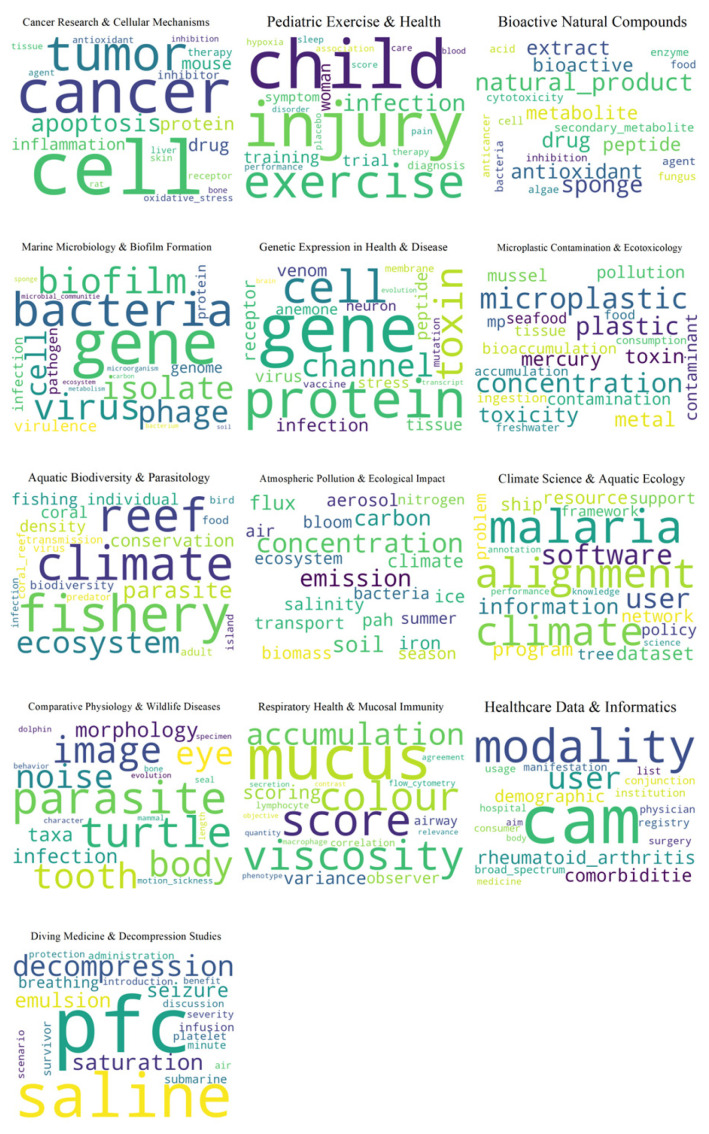
Hierarchical Dirichlet Process model 2016–2023.

Figure 5 displays the Hierarchical Dirichlet Process (HDP) used in the 2016–2023 studies. The IDF model’s clarity and interpretability in topics is again the highest, with a score of 0.57. The figure features word clouds for 13 unique topics complete with titles and descriptions.

The first topic “Climate Science and Aquatic Ecology” focuses on the impact of climate change on aquatic ecosystems and species conservation. The next topic “Microplastic Contamination and Ecotoxicology” assesses the environmental impact of microplastics and other pollutants. “Aquatic Biodiversity and Parasitology” examines the ecological roles of parasites within aquatic environments and implications for biodiversity. “Cancer Research and Cellular Mechanisms” investigates tumor biology, cancer cell apoptosis, and the efficacy of various drug therapies. The next topic “Comparative Physiology and Wildlife Diseases” likely covers comparative studies of physiological structures such as eyes and teeth across different species, including humans and marine life like turtles, with an emphasis on morphological imaging and the study of diseases or infections that impact these species. Alongside the above, “Bioactive Natural Compounds” explores the metabolic effects of bioactive compounds, particularly from marine organisms like sponges. “Genetic Expression in Health and Disease” focuses on researching genetic factors and protein functions, possibly with a neurobiological emphasis. Also, “Pediatric Exercise and Health” focuses on the impact of physical activity on the health and development of children. Additionally, “Marine Microbiology and Biofilm Formation” studies microbial life in marine ecosystems, including the structure and function of biofilms. Furthermore, “Atmospheric Pollution and Ecological Impact” investigates the effects of atmospheric components like aerosols on ecosystems. Subsequently, “Diving Medicine and Decompression Studies” is related to the physiological challenges associated with diving, including decompression sickness. Then, “Healthcare Data and Informatics” utilizes data analysis for healthcare improvement, possibly including demographic and epidemiological studies. Lastly, “Respiratory Health and Mucosal Immunity” explores the role of mucus in respiratory health and immune system defenses.

From 2016 to 2023, the focus moved to using marine science to address global health issues, with a particular focus on respiratory health and the developing field of marine biotechnology. The use of active components originating from marine environments in prevention and treatment techniques has become more diversified. In order to maximize patient care, diagnostic measures grew increasingly complex and data-driven. They also integrated healthcare informatics.

### 3.5. Topic Trend, Status, and Prediction

This section aims to understand and forecast the developments and patterns at the convergence of medical and marine research over three different periods. The analysis’s objectives are to identify the recurrent themes in the collection of publications and track how these themes evolve and intersect over time using the Hierarchical Dirichlet Process model. In addition to analyzing current and previous research directions, the study also aims to predict future trends based on publication growth patterns. The S-curve is utilized to deduce the phases of maturity of various research topics. This makes the analysis both retrospective and predictive. The identification of thematic similarities across the three periods was approached qualitatively, focusing on the commonality of keywords within the topics.

**Figure 6:**
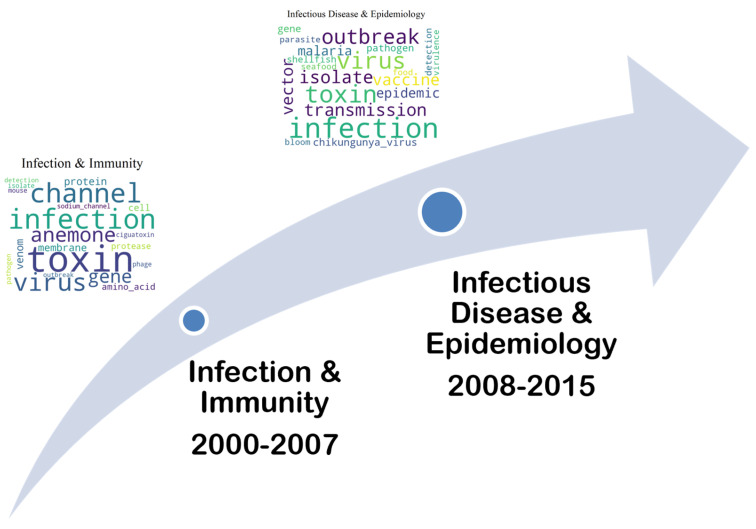
Topic Trend: Infection and Immunity (2000–2007) to Infectious Disease and Epidemiology (2008–2015).

**Figure 7:**
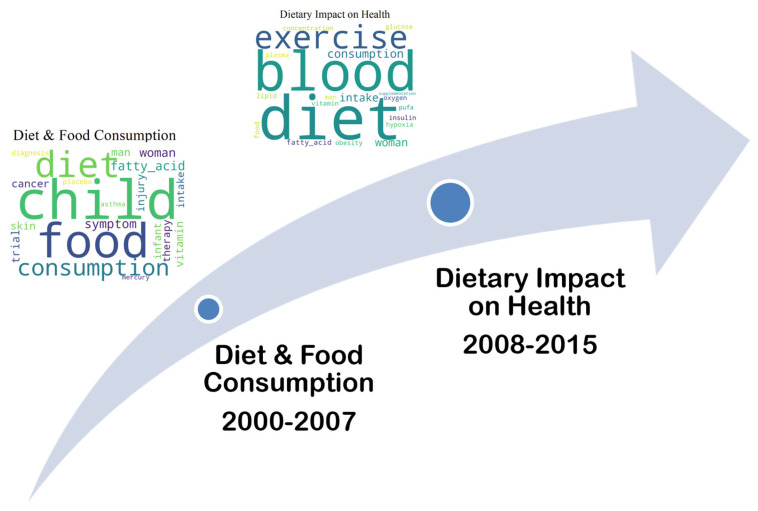
Topic Trend: Diet and Food Consumption (2000–2007) to Dietary Impact on Health (2008–2015).

Figure 6 and Figure 7 illustrate the yearly number of publications for the four topics that did not appear in the third period (2016–2023), with the counts based on the dominant topic identified in each document.

As can be seen in Figure 6, “Infection and Immunity” (2000–2007) seems to evolve into “Infectious Disease and Epidemiology” (2008–2015) in the later period, continuing the focus on disease transmission but with a broader scope, which includes epidemic control. Figure 7 shows that “Diet and Food Consumption” (2000–2007) is closely related to “Dietary Impact on Health” (2008–2015) in the later period, with ongoing research into the health consequences of dietary habits.

**Figure 8:**
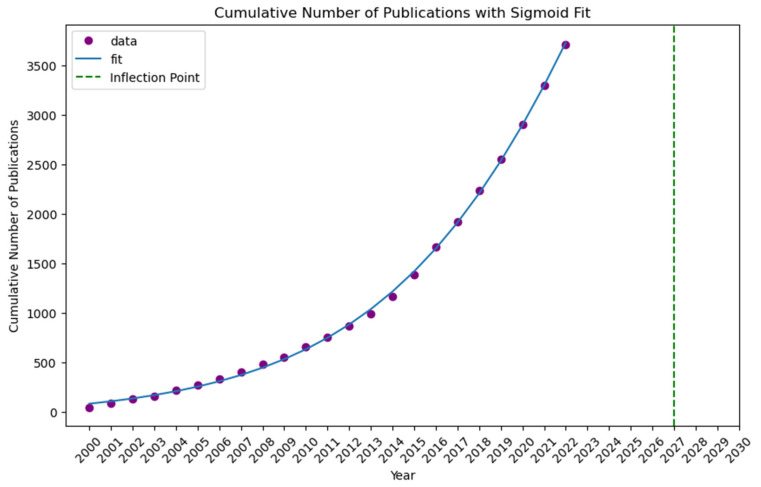

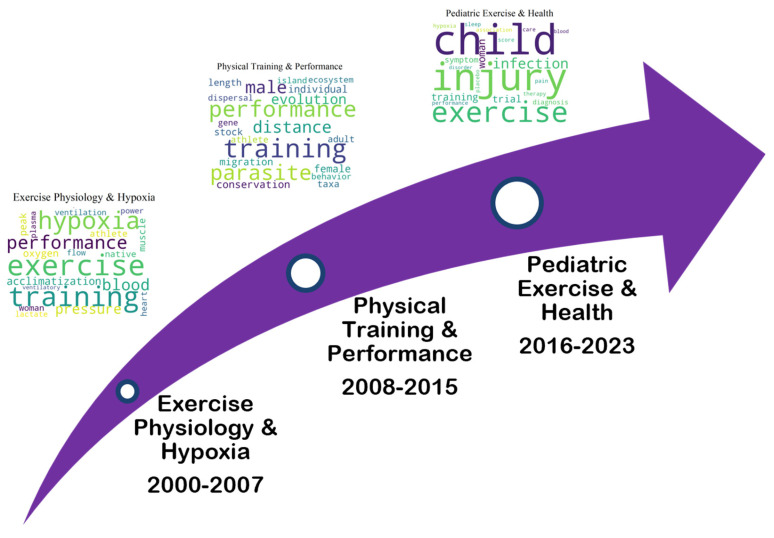
Topic trend, status, and prediction using S-curve-Exercise Physiology and Hypoxia (2000–2007) to Physical Training and Performance (2008–2015) to Pediatric Exercise and Health (2016–2023).

**Figure 9:**
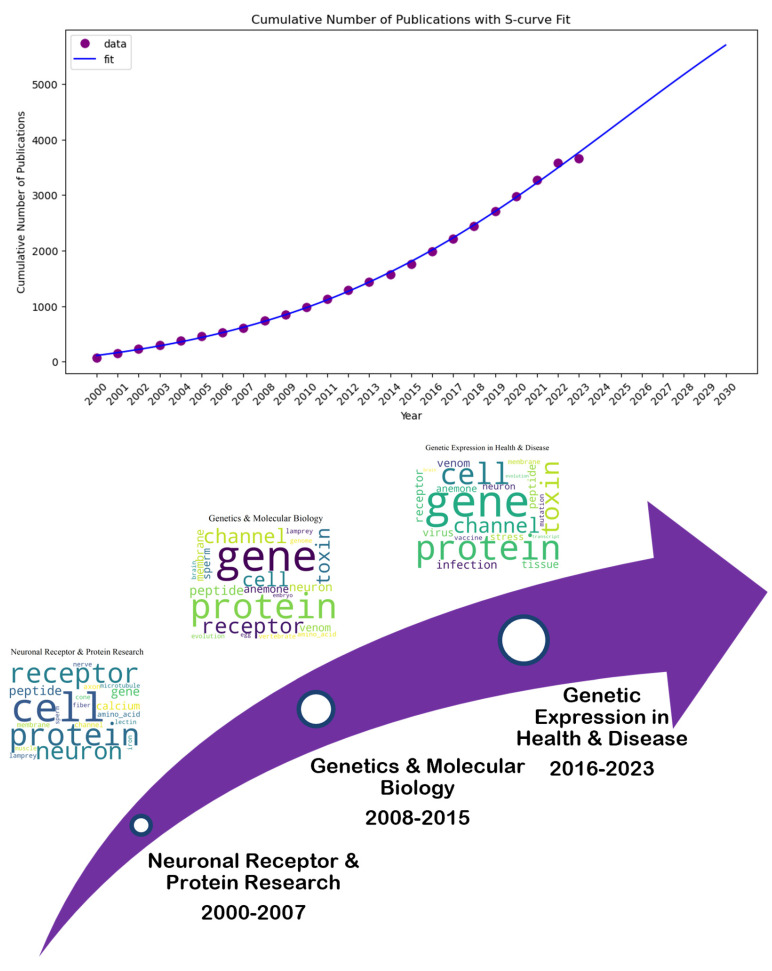
Topic trend, status, and prediction using S-curve-Neuronal Receptor and Protein Research (2000–2007), to Genetics and Molecular Biology (2008–2015), and then to Genetic Expression in Health and Disease (2016–2023).

**Figure 10:**
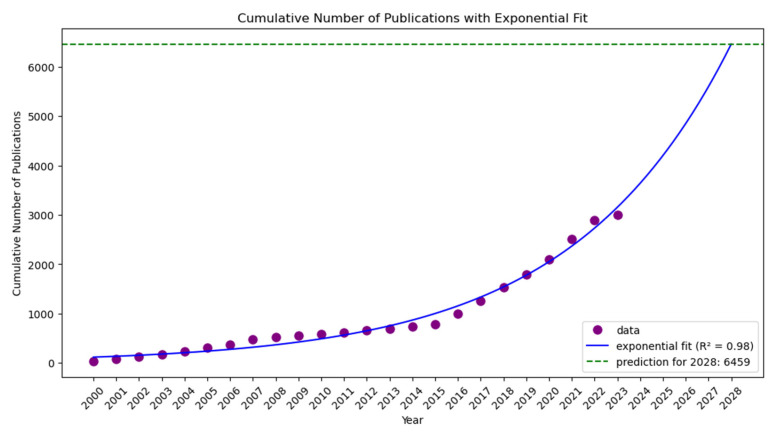

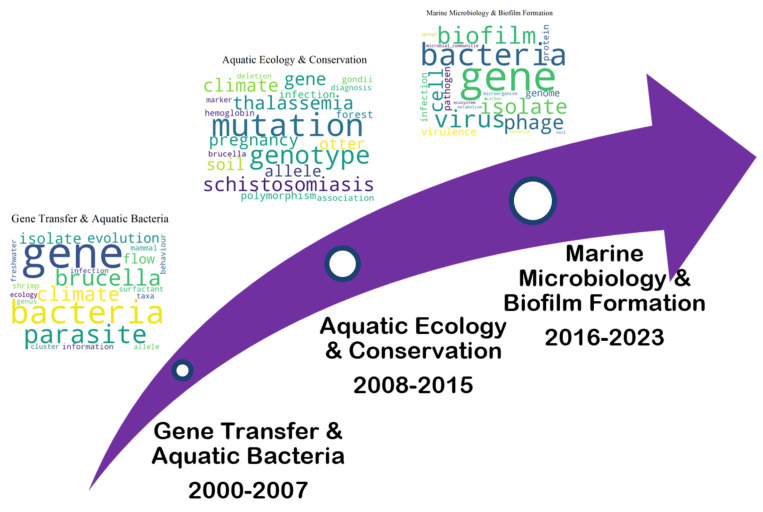
Topic trend, status, and prediction using S-curve-Gene Transfer and Aquatic Bacteria (2000–2007), to Aquatic Ecology and Conservation (2008–2015), and then to Marine Microbiology and Biofilm Formation (2016–2023).

**Figure 11:**
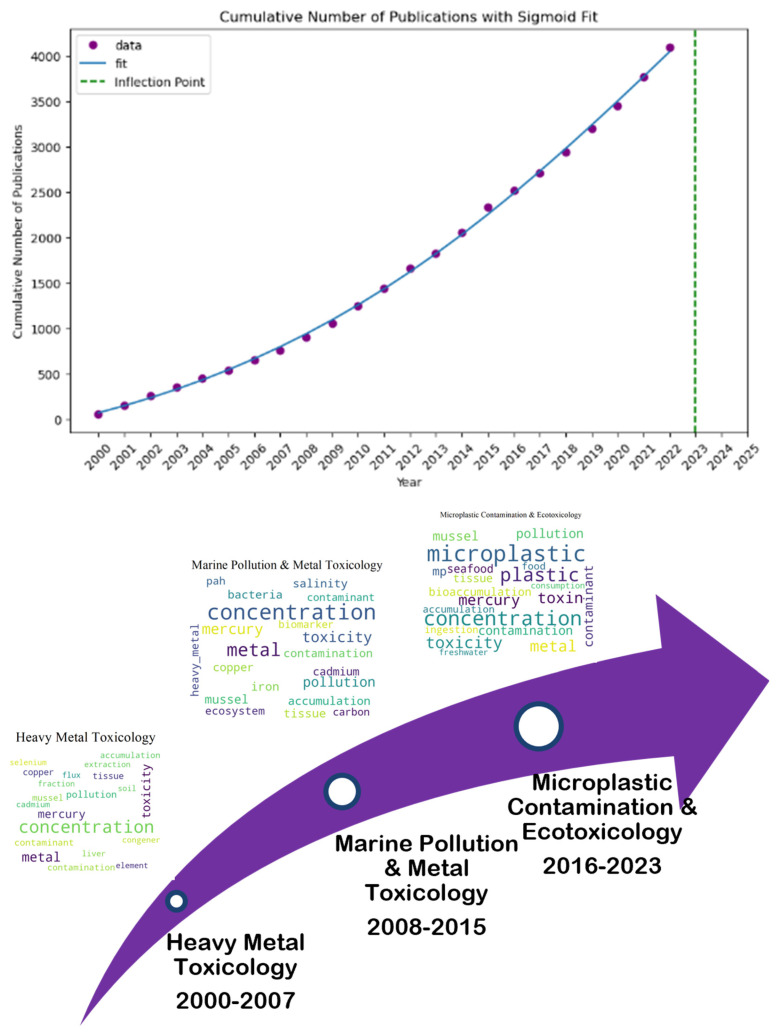
Topic trend, status, and prediction using S-curve-Heavy Metal Toxicology (2000–2007), to Marine Pollution and Metal Toxicology (2008–2015), and then to Microplastic Contamination and Ecotoxicology (2016–2023).

**Figure 12:**
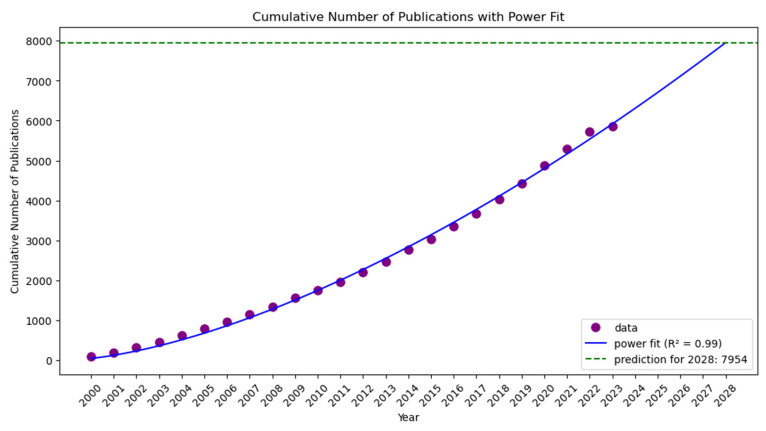

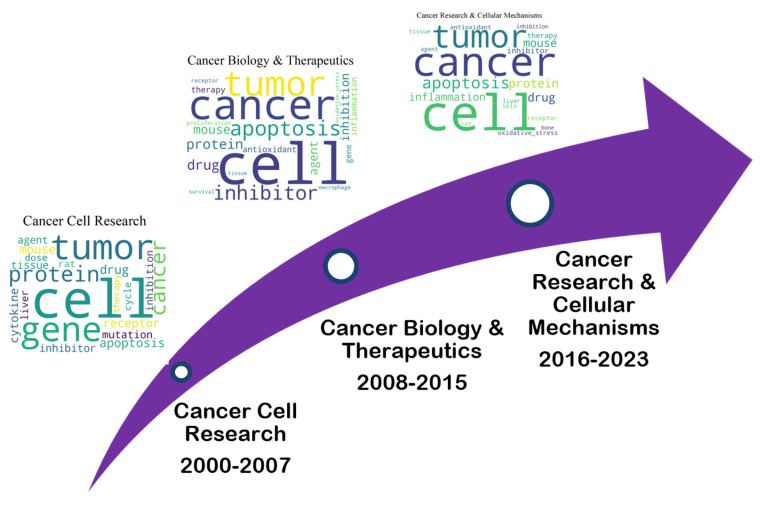
Topic trend, status, Prediction-Cancer Cell Research (2000–2007), to Cancer Biology and Therapeutics (2008–2015), and then to Cancer Research and Cellular Mechanisms (2016–2023).

**Figure 13:**
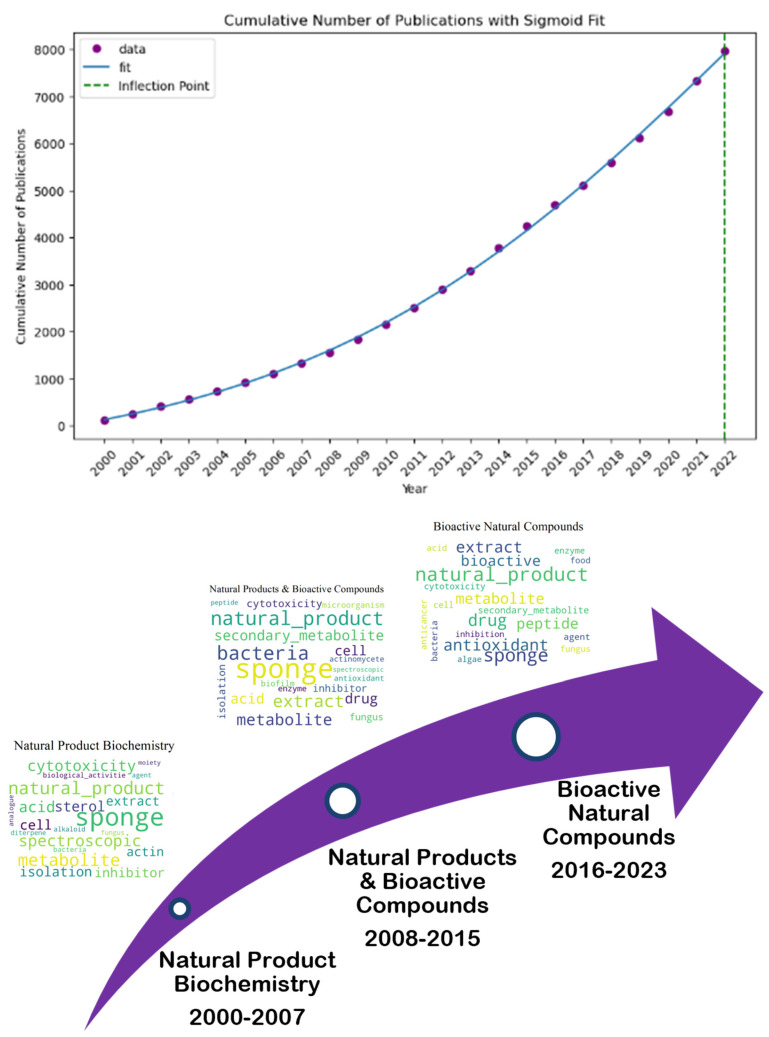
Topic trend, status, and prediction using S-curve-Natural Product Biochemistry (2000–2007), to Natural Products and Bioactive Compounds (2008–2015), and then to Bioactive Natural Compounds (2016–2023).

Conversely, six primary topics have developed across all three periods. This is evident in Figure 8, Figure 9, Figure 10, Figure 11, Figure 12 and Figure 13, each corresponding to one of these evolving topics.

Figure 8 demonstrates that “Exercise Physiology and Hypoxia” (2000–2007) corresponds to “Physical Training and Performance” (2008–2015) in the later period, retaining the core interest in physical health and the body’s response to exercise. Also, it might correspond with “Pediatric Exercise and Health” (2016–2023) in the third period, with a narrowed focus on the pediatric aspect. After combining the three topic names, we chose “Exercise Physiology, Performance, and Pediatric Health” as the suitable one. We then fitted an S-curve to the cumulative number of publications on the topic. Results suggested that the topic is in the growth stage, and the inflection point is believed to be in 2027, with 6305 cumulative publications. An inflection point, which denotes the maximum growth, is the point at which a function shifts from concave to convex or vice versa [58]. However, because making long-term predictions about complex systems is challenging and most likely unreliable [59], we only identify the inflection points within the period until 2030.

This topic highlights that marine science-informed research activities might improve children’s physical performance and health outcomes [60]. Advances in marine biotechnology, such as the creation of nutritional supplements and medicines derived from marine sources, which promote physical performance and recuperation, may be linked to the rise of this research area. New perspectives on health promotion may come from a relationship between marine-based food supplements and physical exercises [61,62,63,64,65,66,67]. Another important point that becomes apparent is the importance of monitoring physiological reactions to exercise, specifically oxygen use and saturation. Especially when considering oxygen dynamics during exertion, it is imperative to incorporate the feature of how exercise affects physiological parameters like VO2 max and SpO2. By including this focus on measurable physical results, the topic’s evolution across time may be seen in a more comprehensive light, thus highlighting the need for accurate measurement in the field of pediatric exercise and health.

Figure 9 presents that “Neuronal Receptor and Protein Research” (2000–2007) continued as “Genetics and Molecular Biology” (2008–2015) with a broader emphasis on genetic expression and molecular processes in neurobiology. Also, it may relate to “Genetic Expression in Health and Disease” (2016–2023) in the third period, maintaining the genetic and neurobiological focus. We determined that “Neuronal and Genetic Mechanisms in Health and Disease” was the best topic name after combining the first three. Next, we fitted an S-curve to the cumulative number of publications related to the subject. According to the results, the topic is still growing, with no inflection point until the year 2030.

This topic represents a promising nexus between medical and marine research, offering insights into neurological health via the genetic variety of marine organisms. Numerous genetic resources from marine creatures have been valuable in the investigation of neural systems. Also, unique insights into the complexity of human neurobiology have been provided by the genetic diversity found in marine life, especially in creatures with complex nervous systems like cephalopods [68]. We predict an increase in marine-derived neurotherapeutics and a deeper investigation of genetic and neurological health as our understanding of these mechanisms advances [69]. As the inflection point will be later than 2030, the field of utilizing genetic methods and insights acquired from marine environments shows an increasing relevance. Novel marine chemicals that may alter genetic and neurological processes linked to diseases like Parkinson’s and Alzheimer’s could be discovered in the future [70,71]. Since multidisciplinary techniques are essential to addressing the complex difficulties at the interface of these fields, collaborations between neurogeneticists and marine scientists are anticipated to gain importance in the future. Through these partnerships, novel biotechnological methods and treatments may be developed, which could revolutionize genetic therapy for neurological illnesses.

As depicted in Figure 10, “Gene Transfer and Aquatic Bacteria” (2000–2007) does not have a direct counterpart in the second period, but the closest in thematic relevance could be “Aquatic Ecology and Conservation” (2008–2015), which also covers genetic aspects within the broader study of marine ecosystems. The biological and ecological mechanisms that underpin each of these subjects’ domains are what link them together. A basic interest in microbial processes influencing broader ecological dynamics may have been indicated by the earlier period’s emphasis on gene transfer within aquatic bacteria. The interest in sustainability naturally spreads to later periods when successful conservation methods depend on an awareness of genetic diversity and the functions that microbes play in ecosystems. As we move from a micro perspective of gene transfer in bacteria to a macro perspective of maintaining biodiversity and ecological integrity within aquatic settings, it is the ecological principles and genetic interactions that give continuity between these themes [72,73,74]. Also, some continuity may be found in “Marine Microbiology and Biofilm Formation” (2016–2023), where microbial life in marine ecosystems is studied, including the structure and function of biofilms in the third period. The name “Aquatic Microbial Dynamics in Conservation, Gene Transfer, and Biofilm Ecology” was the most appropriate after combining the three topic names. The S-curve is not a suitable fit for the data, according to results obtained from fitting the cumulative number of publications to the exponential, linear, logarithmic, power, and S-curve functions. The data are estimated at 0.98 by the exponential function [75], whereas the estimates for the logarithmic, power, and linear functions are 0.55, 0.86, and 0.81, respectively. The R^2^ value is 0.98, and after five years, there have been roughly 6459 publications overall. Therefore, the results do not point to a mid-term saturation of this field’s research activity.

This topic states the critical role that aquatic microbes play in preserving the health of marine environments [76]. Another aspect of this topic is gene transfer in aquatic microorganisms, which provides information on the processes of microbial resistance and the dissemination of genes between species in aquatic environments [77]. Because biofilm ecology is relevant in the medical field, it has attracted a lot of attention. The ecological and evolutionary processes occurring in microbial communities are intricately tied to gene transfer and biofilms. Transfer of bacterial genes is facilitated by biofilms, which behave as complex microecosystems in marine environments. The reason for this is that biofilms provide a stable and concentrated assembly of microbes capable of exchanging genetic material, particularly genes that may enhance survival under specific conditions like resistance to antibiotics [78,79]. Infectious disorders in humans are largely influenced by biofilms, which are populations of bacteria living in a matrix of their own secretion. It is critical to comprehend the dynamics of biofilm formation in order to create new antibacterial methods and control biofilm-associated infections [80]. Furthermore, its long-term strategy suggests that future studies will probably go deeper into the uses of marine microbial research in biotechnology and medicine.

Figure 11 elucidates that “Heavy Metal Toxicology” (2000–2007) continued as “Marine Pollution and Metal Toxicology” (2008–2015), persisting with the theme of environmental contamination and its biological impacts. Also, it may be reflected in “Microplastic Contamination and Ecotoxicology” (2016–2023) in the third period, which deals with the pollution impact of marine ecosystems on health. When the three topic names were combined, “Marine Contaminants and Ecotoxicology Across Heavy Metals and Microplastics” became the most fitting title. After that, an S-curve was fitted again to the cumulative number of articles on the topic. The topic has progressed past the growing stage and has now started to mature according to the data, which show a dropping growth rate. As can be observed, there were 4315 publications overall, with 2023 serving as the potential inflection point.

This topic is an important point of convergence for medical and marine research. An important change in emphasis, from the increasing recognition and identification of marine pollutants to more effectively addressing their effects on the direct and indirect impacts of marine contaminants on public health [81], is suggested by the S-curve analysis. Two of the most important environmental contaminants in maritime ecosystems are known to be heavy metals and microplastics. Because heavy metals cannot biodegrade and have the ability to bioaccumulate, they are extremely dangerous to marine life and, via the food chain, to human health [82]. On the other hand, microplastics have become a worldwide issue because of their prevalence in marine ecosystems, their ability to carry additional pollutants, such as heavy metals, and their potential to harm human health as well as marine life [83]. Furthermore, research indicates that exposure to heavy metals is associated with a number of health problems, such as cancer [84]. The development of this topic shows a thorough approach to solving one of the most important environmental and health issues of our day. Research in this area is anticipated to become practical, transferring scientific knowledge into workable solutions and regulatory frameworks that can lessen the effects of these pollutants on human health and marine ecosystems.

Figure 12 portrays “Cancer Cell Research” (2000–2007) carried on with “Cancer Biology and Therapeutics” (2008–2015), maintaining the focus on tumor biology and extending into therapeutic strategies. Also, it further evolved into “Cancer Research and Cellular Mechanisms” (2016–2023) in the third period, indicating ongoing advancement in cancer research. The most appropriate title was “Cancer Mechanisms and Therapeutic Advances” when the three topic names were combined. Findings from fitting the cumulative number of publications to the exponential, linear, logarithmic, power, and S-curve functions indicate that the S-curve is not a good fit for the data. The estimates for the logarithmic, exponential, and linear functions are 0.94, 0.96, and 0.73, respectively, whereas the power function [85] estimates the data at 0.99. As a matter of fact, the R^2^ value is 0.99, and after five years, the cumulative number of publications is around 7954. As such, the data suggest no mid-term saturation of research activities in this field.

In marine and medical research, this topic is at a pivotal point. The increased fitness of power function in the publication data trend indicates a sustained and growing interest in this field, especially in the investigation of marine-derived components for cancer therapy. Numerous novel bioactive substances with promising anticancer effects have been found in marine creatures. For instance, it has been discovered that substances originating from marine sponges, algae, and microbes demonstrate a variety of anticancer activity mechanisms, including the activation of apoptosis, cell cycle arrest, and blockage of angiogenesis [86,87,88,89,90]. Furthermore, the exploration of marine genomes has opened up new avenues for the research of cancer [91]. The boundary between the marine and medical sciences has also been mentioned in research on the tumor microenvironment, a field in which marine models offer unique insights. Because of their complex interconnections, which mirror the complexity of a tumor, marine ecosystems are excellent models for comprehending the growth and metastasis of cancer [92]. The fact that this field of research is still growing highlights the potential for fresh findings and developments in cancer treatment. The pattern suggests a persistent shift away from basic research and toward more useful therapeutic development.

Finally, as shown in Figure 13, “Natural Product Biochemistry” (2000–2007) progressed into “Natural Products and Bioactive Compounds” (2008–2015), still exploring bioactive marine compounds but perhaps with a refined focus on cellular metabolism. It may correspond with “Bioactive Natural Compounds” (2016–2023) in the third period, suggesting a continued interest in the biochemical potential of natural products. The term “Natural Product Biochemistry and Bioactive Compounds Exploration” was the most appropriate when the three topic names were merged. Following that, the cumulative number of papers on the topic was fitted with an S-curve. The results indicate a declining growth rate. Hence, the topic has moved past the growing stage and is now beginning to mature. As can be seen, the cumulative number of publications was 7968, with 2022 acting as the inflection point.

The development of this topic has been a significant step toward bridging the gap between marine and medical research. S-curve analysis indicates that after 2022, the growth rate of publications will decrease, indicating that this issue has reached a certain level of maturity. The evolution of this field demonstrates a move from discovery to application. Research and development being conducted on bioactive compounds originating from marine sources in order to develop new food supplements and drugs shows this tendency [93]. However, turning the latter findings on pharmaceutical products into practical uses in medicine requires negotiating regulatory processes and proving the products’ effectiveness and safety in demanding clinical studies [94]. This procedure is frequently time-consuming and resource-intensive, which helps to explain why publications have grown at a slower rate recently.

### 3.6. Co-Country Analysis

Our analysis covers 194 countries that are involved in marine medical research. International cooperation in research activities is evident from the average number of collaborations per country, which stands at 37.27. China, with 2987 articles during the past five years, is the most productive country in this global context. China’s expanding impact and dedication to advancing scientific knowledge in this field are highlighted by this impressive output. With 152 unique collaborations, the United States of America is acknowledged as the country with the greatest level of collaboration at the same time.

Important contributions from a wide range of countries were found during our examination of international partnerships in the field of marine medical research. Our study, which employed degree centrality as a critical metric, determined the top 20 countries that have played a major role in advancing this multidisciplinary field’s research ambitions. The top 20 countries together accounted for a sizable share of the world’s research in this multidisciplinary topic. The frequency of collaborations compared to the total number of publications sheds light on the strength and density of these research ties.

**Figure 14:**
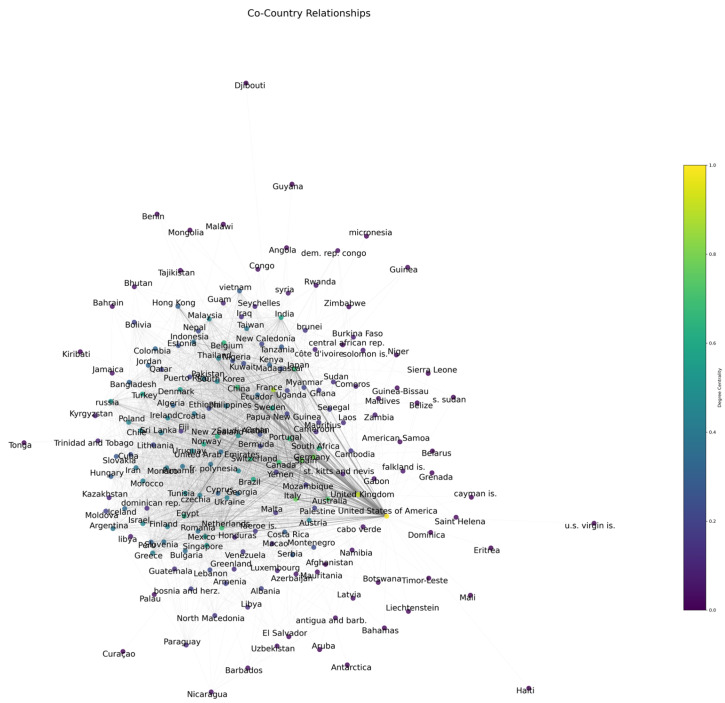
Co-country network based on degree centrality.

As can be seen in Table 2 and Figure 14, the United States of America emerged as the most collaborative country. It has the highest degree of centrality (152), the highest collaboration frequency, and an important position in the worldwide research network. Europe’s prominent role in marine medical research was demonstrated by the United Kingdom and France that followed. With a noteworthy degree centrality of 119 and a sizable number of total publications, Germany, placing fourth, reinforces its position as a prominent contributor. This indicates the nation’s active participation in research relationships.

International relationships have a crucial role in tackling complex global concerns. The United States’ prominent position may be a reflection of its financial support for scientific research as well as its well-established networks [95]. The high degree of centrality of the United States acts as an anchor [96] in the global research network, enabling cross-border collaborations that are critical to addressing challenging problems in marine medicine. These partnership patterns have significant consequences for future funding and policy initiatives targeted at fostering international scientific cooperation in addition to being illustrative of current research dynamics. The synergy between countries with high degrees of centrality and high frequency of collaboration is going to progress in areas like understanding the effects of changes in the marine environment on human health and developing drugs from marine natural products.

### 3.7. Co-Organization Analysis

**Figure 15:**
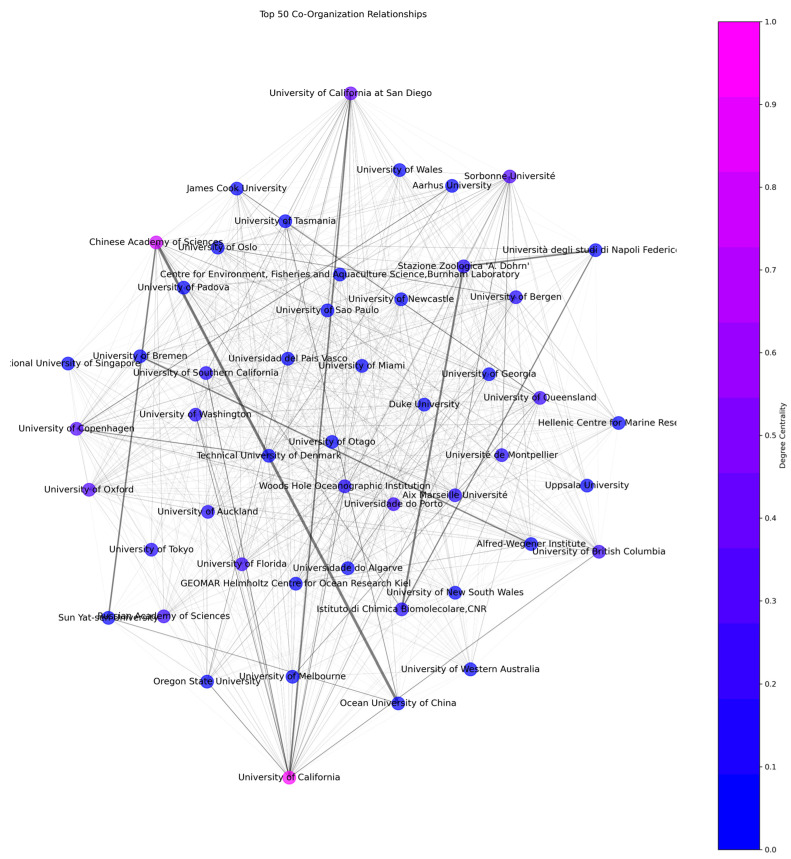
Co-organization network based on degree centrality.

The vast network of institutions engaged in marine medical research is demonstrated by the analysis we conducted, which includes 18,097 organizations in total, with 12.9 as the average number of collaborations per organization. A collaborative research culture is largely fostered by the University of California, which stands out as the most collaborative organization with an astounding 1103 scientific collaborations in the field of investigation. The university’s dedication to creating and sustaining strong research networks is reflected in this degree of involvement [97]. Degree centrality represents the number of unique collaborations each organization has. Collaboration frequency is calculated as the sum of the frequencies of all collaborations for each organization. As evidenced by Figure 15, as well as Table 3 and Table 4, its remarkable 2067 collaborations put the University of California at the top, demonstrating its wide-ranging involvement in science. In second place, the Chinese Academy of Sciences has a degree centrality of 891 and 2402 instances of collaboration, highlighting its great interconnectedness and 1331 publications of research output. With 587 publications during the last five years, the Chinese Academy of Sciences has recently become the most productive organization. Another indication of the noteworthy contribution of California’s universities to the area is the notable appearance of the University of California, San Diego, which has a degree centrality of 647 and a collaboration frequency of 1198. Notable actors include the Sorbonne Université, which stands out for its significant position within the research network with a degree centrality of 590 and a collaboration frequency of 1016.

The Chinese Academy of Sciences is identified as a crucial intermediate with a score of 0.055 when betweenness centrality within the network of collaborations in marine medical research is examined. With a betweenness centrality value of 0.052, the University of California is also clearly important. With scores of 0.038 and 0.025, respectively, the Universities of California, San Diego, and Queensland also exhibit noteworthy connective significance, serving as essential nodes in the network of international research collaborations. The high betweenness centrality of the Chinese Academy of Sciences, being the mainstay of the marine medical research network, and the University of California underscores their dual position as the major bridges that promote cooperation between different organizations. As demonstrated by their high degree and betweenness centrality scores, the University of Queensland, the University of California at San Diego, and Sorbonne Université are also major participants within the global research network. Their strategic positions imply that they are not only important contributors but also important facilitators of joint research initiatives. Innovation and discovery are accelerated by the central positions these institutions hold within the network, where they facilitate the exchange of resources and knowledge. Fostering international collaboration and innovation in the marine medical area is heavily reliant on the interconnection of research institutions and the global dispersion of countries [98].

### 3.8. Co-Author Analysis

**Figure 16:**
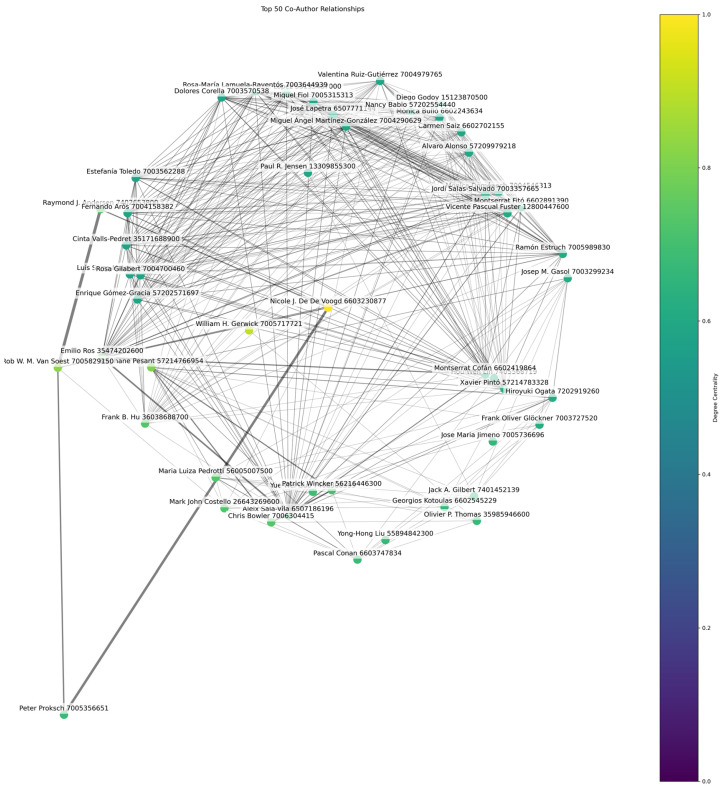
Co-author network based on degree centrality.

A total of 142,681 authors are included in the co-authorship analysis of marine medical research. The scholars exhibit a dynamic and integrated academic community, as seen by an average of 10.57 collaborations per author. As demonstrated by Figure 16, along with Table 5 and Table 6, Nicole J. De Voogd is the author with the greatest number of collaborations (403), indicating a wide network of co-authored research on the subject. Also, her collaboration frequency of 629 indicates that she is the most central author in marine medical research. Furthermore, Nicole J. De Voogd ranks highest in terms of betweenness centrality (0.022), indicating the critical involvement in bridging research clusters.

The next most connected person in the community is William H. Gerwick, with a degree centrality of 365 and 683 partnerships. Additionally, noteworthy researchers are Rob W. M. Van Soest and Stéphane Pesant, whose high collaboration and centrality counts highlight their significant roles within the network. The intensity of cooperation indicates a high preference for multidisciplinary research and teamwork. The result illustrates a notable individual influence on the amount of research conducted in the field of marine medicine, which suggests that key individuals have an impact on scholarly trends and the creation of fresh lines of inquiry [99,100]. The collaborative character of these efforts frequently crosses academic boundaries, promoting inventive methods for resolving intricate issues at the intersection of medical and marine biology [101,102,103]. So, the strategic support of individuals who are critical nodes in the research network is imperative as these researchers not only promote knowledge but also define funding priorities and research policies [104].

## 4. Implications for Research Policy and Management

Our quantitative analysis of the existing literature at the intersection of marine sciences and medical applications highlights the value of text mining methods for analyzing scientific trends and identifying future areas of focus for research and development. Research organizations, innovative companies, and R&D policymakers should incorporate such methods into their strategic planning toolkit. Although, as demonstrated below, this type of analysis can only provide a portion of the informational foundation, the results of the bibliometric analysis yield concrete implications. Research organizations and initiatives dedicated to the interface between marine research and medical research can utilize the results to derive important implications for their own research policy and their strategic management of research and development. Depending on their own reputation and resource strength, they can focus on the dominant themes and most central organizations and research actors, respectively. Alternatively, niche strategies can be chosen, which may more realistically reflect their own capabilities. Based on our findings, we can provide the following implications for future research at the interface between marine and medical research:

Strategic Focus Areas: Marine research on “Aquatic Microbial Dynamics in Conservation, Gene Transfer, and Biofilm Ecology” and “Natural Product Biochemistry and Bioactive Compounds Exploration” may have implications for health research, especially in terms of creating novel treatments. Research institutions might investigate how to use this research to develop treatments for infectious diseases. Both topics are of interest in terms of health applications as microbes play important roles in both the environment and human health. Aquatic microorganisms contribute to nutrient cycling, pollutant degradation, and the health of marine ecosystems, and they indirectly affect human health. Furthermore, understanding the mechanisms of gene transfer among aquatic microbes, which often include antibiotic resistance, could lead to the development of new antibiotics and treatments for bacterial infections. Such studies of microbial dynamics can provide strategies against infectious diseases, an important health challenge.

International Partnerships: Since the United States and other countries are highly central in international collaborations, research organizations may establish or strengthen strategic partnerships with international partners. Results from these collaborations could include sharing best practices, increasing awareness within the scientific community, and creating more networking opportunities.

Emphasize High-Impact Collaborations: Cutting-edge research facilities and technologies are necessary for performing cutting-edge marine medical science research, and collaborations with prominent organizations may provide access to these. Collaborations with such institutions provide access to advanced technology and knowledge. The Chinese Academy of Sciences is one example of an entity with high betweenness centrality that is essential to linking different clusters within the network. One advantage of working with such organizations is that researchers can obtain direct access to many sub-networks and increase the effect of research findings.

Interact with Key Authors: Researchers with high collaboration frequency, such as William H. Gerwick or Nicole J. de Voogd, are likely to have great influence and large networks. Getting involved with these researchers in advisory committees or cooperative research projects can be advantageous. Also, certain researchers have demonstrated a high degree of centrality and the overall quantity of publications. The scientific impact and visibility of research could be enhanced by collaborating with these individuals on collaborative publications and projects.

Optimizing Collaboration through Strategic Management: Authors who possess a high betweenness centrality are prone to establishing connections between different clusters within the research network. By working together with these researchers, the research organization may be positioned as the network’s hub. The strategic management of the collaboration network helps to avoid drawbacks, such as large resource demands, potential intellectual property problems, and challenges arising from different organizational cultures. It would also allow for the setting up of regulations that secure the sharing of sensitive data and create trust. Addressing these obstacles is critical for reducing risks and optimizing the advantages of collaborations in marine medical research.

Research initiatives, such as the BlueHealthTech R&D program, have the opportunity to leverage strategic insights to guide the medium-term direction of their research activities. By aligning their priorities with emerging trends and interdisciplinary demands, they can effectively position themselves as key contributors to the rapidly evolving field at the interface of marine sciences and medical applications. The systematic derivation of research and development priorities not only enhances the strategic focus of such initiatives but also builds trust among relevant stakeholders, including research policy supporters and potential cooperation partners. This transparency fosters a sense of shared purpose, enabling stakeholders to converge on common objectives and optimally allocate limited resources. By doing so, initiatives like BlueHealthTech can drive synergies that amplify the impact of their efforts, even in the face of constrained funding and capacity. R&D topics at the intersection of marine sciences and medical applications inherently involve a high degree of uncertainty, driven by the complexity of the fields and the nascent state of certain technological and scientific advances. As such, enhanced foresight mechanisms, including scenario planning, trend analysis, and stakeholder consultation, are critical. These tools empower stakeholders across the innovation ecosystem to anticipate challenges, identify opportunities, and build actionable capacity. By fostering a forward-looking mindset, BlueHealthTech can cultivate the motivation and readiness necessary to tackle these complex, interdisciplinary challenges effectively. The analysis underscores the imperative to systematically build collaborative networks that span disciplines, sectors, and institutions. Strengthening existing capacities for cross-disciplinary collaboration is essential for accelerating innovation and translating scientific discoveries into practical applications. Networks that integrate expertise from marine biology, oceanography, medical science, biotechnology, and related fields can unlock novel solutions to pressing health and environmental challenges, ranging from marine-derived pharmaceuticals to strategies for mitigating health risks posed by marine pollution. To this end, research initiatives like BlueHealthTech should not only serve as hubs of innovation but also as educational enablers. By supporting individual researchers and teams through targeted training programs, fellowships, and skill-building workshops, they can prepare the next generation of scientists to operate effectively at the nexus of marine and medical sciences. These educational initiatives should emphasize interdisciplinary thinking, practical problem-solving, and collaboration across traditional academic and industrial boundaries. Ultimately, BlueHealthTech’s role in shaping R&D priorities, fostering trust among stakeholders, and cultivating interdisciplinary expertise can significantly enhance the innovation ecosystem. By systematically addressing the uncertainties and capacity gaps inherent in this field, BlueHealthTech can position itself as a leader in driving impactful research, creating actionable outcomes, and contributing to global health and sustainable ocean stewardship.

## 5. Limitations and Future Research

Although this study provides a wide overview of the rising convergence of marine and medical research, it is necessary to recognize a few limitations. First, our reliance on a single database (Scopus) may not convey the breadth of global research efforts. Furthermore, much subjective judgment was still required to assess research issues even when sophisticated methods such as Hierarchical Dirichlet Process modeling were used. This implies that some subtle, developing themes or early-stage partnerships, which have not resulted in official publications yet, may not be as well-represented. Lastly, while we forecasted future changes based on past data, funding priorities and science can change suddenly. Unpredictably, breakthrough discoveries, policy changes, or global health issues may shift the course of this subject. For example, in the course of the COVID-19 pandemic, global health research has focused primarily on this critical issue at the expense of other health issues [3].

These limits show the promising steps for the future. Additional databases such as Medline or emerging literature, as well as patents and industry reports, may be used in future research to present a more thorough picture. In addition, a deeper inquiry through an enhanced analysis of the full texts available would allow us to provide more concrete recommendations for research practice. Also, future research should consider environmental changes, regulatory changes, and economic conditions affecting these emerging fields to identify how external factors shape the research and development landscape. Furthermore, assessing the quality of articles using measures such as citation counts and journal impact factors can provide a more complete picture of the field’s progress. Our analysis identified relevant research articles based on the co-occurrence of marine and medical terms without taking into account qualitative information about the intensity of such convergence. Future research could complement this analysis by using more advanced deep learning methods to provide additional information about the topics of the two distant scientific fields that overlap more intensely over time [105].

Using focus groups, workshops, or interviews to speak with researchers and business leaders directly would provide important context for the trends we have seen. Such a qualitative analytical approach may allow for the discovery of hidden connections and the identification of early signals of evolving themes. Engaging in focus groups, interviews, or workshops with these specialists can offer important qualitative data regarding the challenges, motivations, and contexts related to this subject. If we broaden our sources and adopt different methodologies, we will be in an even better position to understand how marine science and the medical sciences will even more positively affect each other in the future. This approach can yield additional valuable insights for academics, policymakers, and organizations. Policymakers might use these insights to better allocate resources and promote innovation in critical sectors like drug research and sustainable health solutions. These projects would strengthen the scientific base of marine medical research while simultaneously increasing its societal impact.

## 6. Conclusions

In summary, the analysis shows the high complexity of research at the intersection of marine science and medicine. The topics enabled by the convergence of these two fields are very diverse and range from the use of marine organisms to the mutual application of scientific methods. The increasing number of publications and the breadth of identified interfaces demonstrate the relevance of collaboration between these previously often separately considered scientific fields. At the same time, there is a need for an in-depth analysis of research trends and the identification of relevant research topics, as well as cooperation partners with expertise from both research areas. This enables the interpretation of research trends and allows the consideration of the competencies and infrastructures available at the respective research institutes. Research strategies are always, to some extent, emergent, i.e., they develop from existing scientific expertise and from the results of previous projects. The task of strategic research and development planning is to reconcile both perspectives: the top–down prioritization of research topics and cooperation partners, with the bottom–up view of utilizing existing competencies and experiences.

## Figures and Tables

**Table 1:** Top 20 trends in journal subjects according to WoS categories based on frequency (2000–2023).

Row	2000–2007			2008–2015			2016–2023		
	WOS Category	Count	(Count/Total) × 100	WOS Category	Count	(Count/Total) × 100	WOS Category	Count	(Count/Total) × 100
1	Pharmacology and Pharmacy	105	7.692	Pharmacology and Pharmacy	148	6.871	Pharmacology and Pharmacy	159	6.257
2	Neurosciences	60	4.396	Neurosciences	97	4.503	Medicine, General and Internal	106	4.172
3	Immunology	53	3.883	Medicine, General and Internal	85	3.946	Public, Environmental, and Occupational Health	103	4.054
4	Medicine, General and Internal	47	3.443	Clinical Neurology	65	3.018	Neurosciences	99	3.896
5	Toxicology	41	3.004	Oncology	63	2.925	Oncology	79	3.109
6	Biochemistry and Molecular Biology	40	2.930	Immunology	62	2.878	Biochemistry and Molecular Biology	65	2.558
7	Clinical Neurology	40	2.930	Public, Environmental, and Occupational Health	62	2.878	Clinical Neurology	65	2.558
8	Oncology	38	2.784	Biochemistry and Molecular Biology	57	2.646	Immunology	63	2.479
9	Public, Environmental, and Occupational Health	37	2.711	Psychiatry	56	2.600	Microbiology	62	2.440
10	Endocrinology and Metabolism	35	2.564	Infectious Diseases	54	2.507	Toxicology	61	2.401
11	Infectious Diseases	30	2.198	Microbiology	53	2.461	Infectious Diseases	60	2.361
12	Microbiology	30	2.198	Toxicology	52	2.414	Medicine, Research and Experimental	57	2.243
13	Cell Biology	29	2.125	Cardiac and Cardiovascular System	49	2.275	Psychiatry	56	2.204
14	Chemistry, Medicinal	28	2.051	Endocrinology and Metabolism	47	2.182	Endocrinology and Metabolism	48	1.889
15	Nutrition and Dietetics	27	1.978	Chemistry, Medicinal	43	1.996	Chemistry, Medicinal	46	1.810
16	Surgery	25	1.832	Medicine, Research and Experimental	41	1.903	Pediatrics	46	1.810
17	Dermatology	24	1.758	Nutrition and Dietetics	41	1.903	Surgery	46	1.810
18	Genetics and Heredity	24	1.758	Dermatology	38	1.764	Cardiac and Cardiovascular System	44	1.732
19	Medicine, Research and Experimental	24	1.758	Genetics and Heredity	38	1.764	Nutrition and Dietetics	44	1.732
20	Cardiac and Cardiovascular System	23	1.685	Surgery	36	1.671	Dermatology	40	1.574
**Total**		1365		Total	2154		Total	2541	
**Articles in journals not indexed in WOS**		471		Articles in journals not indexed in WOS	817		Articles in journals not indexed in WOS	888	

**Table 2:** Top 20 countries based on their degree centrality, collaboration frequency, and total number of publications.

Row	Country	Degree Centrality	Collaboration Frequency	Total Number of Publications	Collaboration Frequency/Total Number of Publications
1	United States of America	152	6796	8557	0.79
2	United Kingdom	139	3850	2483	1.55
3	France	134	3109	2299	1.35
4	Germany	119	3414	2462	1.39
5	Spain	118	2759	2261	1.22
6	Italy	117	2257	2269	0.99
7	Australia	110	2196	1841	1.19
8	Canada	107	1955	1495	1.31
9	Netherlands	104	1460	805	1.81
10	China	103	2485	5919	0.42
11	Japan	102	1857	2820	0.66
12	Belgium	100	1005	481	2.09
13	Portugal	99	1139	903	1.26
14	Switzerland	99	1127	540	2.09
15	South Africa	97	734	330	2.22
16	New Zealand	95	818	554	1.48
17	Egypt	92	1021	766	1.33
18	Brazil	91	997	1189	0.84
19	Norway	90	1284	900	1.43
20	Sweden	88	1114	607	1.84

**Table 3:** Top 20 organizations based on their degree centrality, collaboration frequency, and total number of publications.

Row	Organization	Degree Centrality	Collaboration Frequency	Total Number of Publications	Collaboration Frequency/Total Number of Publications
1	University of California	1103	2067	654	3.16
2	Chinese Academy of Sciences	891	2402	1331	1.80
3	University of California at San Diego	647	1198	431	2.78
4	Sorbonne Université	590	1016	207	4.91
5	University of Copenhagen	523	825	223	3.70
6	University of Oxford	518	719	155	4.64
7	Universidade do Porto	515	1019	340	3.00
8	University of Queensland	514	798	302	2.64
9	Russian Academy of Sciences	477	1044	654	1.60
10	University of Florida	474	687	202	3.40
11	University of British Columbia	450	790	246	3.21
12	Aix Marseille Université	441	635	150	4.23
13	University of Washington	429	634	180	3.52
14	Université de Montpellier	409	588	140	4.20
15	University of Bergen	408	648	171	3.79
16	Stazione Zoologica ‘A. Dohrn’	408	788	227	3.47
17	University of Auckland	385	609	151	4.03
18	University of Tokyo	382	775	337	2.30
19	Woods Hole Oceanographic Institution	379	600	149	4.03
20	Istituto di Chimica Biomolecolare, CNR	376	684	264	2.59

**Table 4:** Top 20 organizations based on betweenness centrality.

Row	Organization	Betweenness Centrality
1	Chinese Academy of Sciences	0.055
2	University of California	0.052
3	University of California at San Diego	0.038
4	University of Queensland	0.025
5	Russian Academy of Sciences	0.024
6	University of Copenhagen	0.021
7	University of British Columbia	0.02
8	Duke University	0.02
9	French Research Institute for Exploitation of the Sea (IFREMER)	0.019
10	Universidad Nacional Autónoma de México	0.018
11	Pukyong National University	0.017
12	Hokkaido University	0.015
13	King Saud University	0.014
14	Universidade do Porto	0.014
15	Université Pierre et Marie Curie	0.013
16	Universidad del Pais Vasco	0.013
17	Aix Marseille Université	0.013
18	Nagasaki University	0.013
19	Sorbonne Université	0.013
20	Korea Ocean Research and Development Institute	0.013

**Table 5:** Top 20 authors based on their degree centrality, collaboration frequency, and total number of publications.

Row	Author with Scopus Author ID	Degree Centrality	Collaboration Frequency	Total Number of Publications	Collaboration Frequency/Total Number of Publications
1	Nicole J. De De Voogd 6603230877	403	629	64	9.83
2	William H. Gerwick 7005717721	365	683	109	6.27
3	Rob W. M. Van Soest 7005829150	334	520	77	6.75
4	Stéphane Pesant 57214766954	325	404	7	57.71
5	Emilio Ros 35474202600	308	348	14	24.86
6	Maria Luiza Pedrotti 56005007500	296	303	4	75.75
7	Frank B. Hu 36038688700	296	313	11	28.45
8	Chris Bowler 7006304415	295	395	18	21.94
9	Raymond J. Andersen 7402653800	292	421	52	8.10
10	Mark John Costello 26643269600	291	293	7	41.86
11	Patrick Wincker 56216446300	284	420	19	22.11
12	Yue-Wei Guo 7406308399	276	622	122	5.10
13	Aleix Sala-Vila 6507186196	274	307	9	34.11
14	Pascal Conan 6603747834	274	308	8	38.50
15	Peter Proksch 7005356651	273	600	102	5.88
16	Olivier P. Thomas 35985946600	272	399	54	7.39
17	Georgios Kotoulas 6602545229	270	287	8	35.88
18	Yong-Hong Liu 55894842300	270	608	80	7.60
19	Jose Maria Jimeno 7005736696	266	428	54	7.93
20	Jack A. Gilbert 7401452139	263	266	5	53.20

**Table 6:** Top 20 authors based on betweenness centrality.

Row	Author with Scopus Author ID	Betweenness Centrality
1	Nicole J. De De Voogd 6603230877	0.022
2	William H. Gerwick 7005717721	0.014
3	Rob W. M. Van Soest 7005829150	0.014
4	Derek C. G. Muir 7202872916	0.012
5	Frank B. Hu 36038688700	0.012
6	Frances M. D. Gulland 7006272482	0.011
7	Vincenzo Di Di Marzo 7101602863	0.01
8	Michael G. Ziegler 7202530437	0.009
9	Paul Kwan-Sing Lam 7202365776	0.009
10	Shinsuke Tanabe 7401677757	0.008
11	Olivier P. Thomas 35985946600	0.008
12	Serge Planes 7004574648	0.008
13	Nikki A. Ford 25651627600	0.007
14	Chang-Yun Wang 7501631599	0.007
15	Marie-Lise Bourguet-Kondracki 6602732915	0.007
16	Peter S. Ross 7402412702	0.007
17	Ursula Siebert 7004796609	0.007
18	Rainer Lohmann 7007118339	0.006
19	De-Hai Li 14422493800	0.006
20	Curtis A. Suttle 7004913800	0.006

## Data Availability

The data supporting the results of this study are available in publicly accessible repositories indexed in Scopus. For further details, please contact the corresponding author.

## Appendix A

**Table A1:** Trends in journal subjects according to WoS categories based on frequency (2000–2023).

2000–2007		
WOS Categories	Count	(Count/Total) × 100
**Pharmacology and Pharmacy**	105	7.692
**Neurosciences**	60	4.396
**Immunology**	53	3.883
**Medicine, General and Internal**	47	3.443
**Toxicology**	41	3.004
**Biochemistry and Molecular Biology**	40	2.930
**Clinical Neurology**	40	2.930
**Oncology**	38	2.784
**Public, Environmental, and Occupational Health**	37	2.711
**Endocrinology and Metabolism**	35	2.564
**Infectious Diseases**	30	2.198
**Microbiology**	30	2.198
**Cell Biology**	29	2.125
**Chemistry, Medicinal**	28	2.051
**Nutrition and Dietetics**	27	1.978
**Surgery**	25	1.832
**Dermatology**	24	1.758
**Genetics and Heredity**	24	1.758
**Medicine, Research and Experimental**	24	1.758
**Cardiac and Cardiovascular System**	23	1.685
**Sport Sciences**	21	1.538
**Physiology**	19	1.392
**Psychiatry**	19	1.392
**Obstetrics and Gynecology**	18	1.319
**Pathology**	18	1.319
**Radiology, Nuclear Medicine and Medical Imaging**	18	1.319
**Hematology**	17	1.245
**Pediatrics**	17	1.245
**Respiratory System**	17	1.245
**Biology**	16	1.172
**Peripheral Vascular Diseases**	16	1.172
**Environmental Sciences**	14	1.026
**Ophthalmology**	14	1.026
**Allergy**	13	0.952
**Parasitology**	13	0.952
**Chemistry, Multidisciplinary**	12	0.879
**Reproductive Biology**	12	0.879
**Urology and Nephrology**	12	0.879
**Tropical Medicine**	11	0.806
**Biochemical Research Methods**	10	0.733
**Critical Care Medicine**	10	0.733
**Behavioral Sciences**	9	0.659
**Biotechnology and Applied Microbiology**	9	0.659
**Dentistry, Oral Surgery and Medicine**	9	0.659
**Emergency Medicine**	9	0.659
**Gastroenterology and Hepatology**	9	0.659
**Psychology**	9	0.659
**Rehabilitation**	9	0.659
**Biophysics**	8	0.586
**Chemistry, Analytical**	8	0.586
**Food Science and Technology**	8	0.586
**Health Care Sciences and Services**	8	0.586
**Medical Laboratory Technology**	8	0.586
**Rheumatology**	8	0.586
**Chemistry, Organic**	7	0.513
**Engineering, Biomedical**	7	0.513
**Plant Sciences**	7	0.513
**Psychology, Experimental**	7	0.513
**Virology**	7	0.513
**Orthopedics**	6	0.440
**Veterinary Sciences**	6	0.440
**Anesthesiology**	5	0.366
**Developmental Biology**	5	0.366
**Mathematical and Computational Biology**	5	0.366
**Medicine, Legal**	5	0.366
**Transplantation**	5	0.366
**Zoology**	5	0.366
**Chemistry, Applied**	4	0.293
**Health Policy and Services**	4	0.293
**Integrative and Complementary Medicine**	4	0.293
**Mycology**	4	0.293
**Nuclear Science and Technology**	4	0.293
**Nursing**	4	0.293
**Otorhinolaryngology**	4	0.293
**Psychology, Biological**	4	0.293
**Anatomy and Morphology**	3	0.220
**Computer Science, Artificial Intelligence**	3	0.220
**Geriatrics and Gerontology**	3	0.220
**Medical Informatics**	3	0.220
**Microscopy**	3	0.220
**Primary Health Care**	3	0.220
**Psychology, Clinical**	3	0.220
**Anthropology**	2	0.147
**Computer Science, Interdisciplinary Applications**	2	0.147
**Ecology**	2	0.147
**Evolutionary Biology**	2	0.147
**Gerontology**	2	0.147
**Marine and Freshwater Biology**	2	0.147
**Multidisciplinary Sciences**	2	0.147
**Neuroimaging**	2	0.147
**Psychology, Development**	2	0.147
**Substance Abuse**	2	0.147
**Acoustics**	1	0.073
**Andrology**	1	0.073
**Audiology and Speech-Language Pathology**	1	0.073
**Cell and Tissue Engineering**	1	0.073
**Chemistry, Inorganic and Nuclear**	1	0.073
**Chemistry, Physical**	1	0.073
**Education and Educational Research**	1	0.073
**Education, Special**	1	0.073
**Engineering, Chemical**	1	0.073
**Engineering, Multidisciplinary**	1	0.073
**Entomology**	1	0.073
**Environmental Studies**	1	0.073
**Ethics**	1	0.073
**Fisheries**	1	0.073
**History and Philosophy Of Science**	1	0.073
**Information Science and Library Science**	1	0.073
**Management**	1	0.073
**Materials Science, Biomaterials**	1	0.073
**Mathematics, Interdisciplinary Applications**	1	0.073
**Oceanography**	1	0.073
**Psychology, Applied**	1	0.073
**Robotics**	1	0.073
**Social Sciences, Mathematical Methods**	1	0.073
**Social Work**	1	0.073
**Spectroscopy**	1	0.073
**Statistics and Probability**	1	0.073
**Water Resources**	1	0.073
**Total**	1365	
**These journals are not indexed in WOS**	471	
**2008–2015**		
**WOS Categories**	**Count**	**(Count/Total) × 100**
**Pharmacology and Pharmacy**	148	6.871
**Neurosciences**	97	4.503
**Medicine, General and Internal**	85	3.946
**Clinical Neurology**	65	3.018
**Oncology**	63	2.925
**Immunology**	62	2.878
**Public, Environmental, and Occupational Health**	62	2.878
**Biochemistry and Molecular Biology**	57	2.646
**Psychiatry**	56	2.600
**Infectious Diseases**	54	2.507
**Microbiology**	53	2.461
**Toxicology**	52	2.414
**Cardiac and Cardiovascular System**	49	2.275
**Endocrinology and Metabolism**	47	2.182
**Chemistry, Medicinal**	43	1.996
**Medicine, Research and Experimental**	41	1.903
**Nutrition and Dietetics**	41	1.903
**Dermatology**	38	1.764
**Genetics and Heredity**	38	1.764
**Surgery**	36	1.671
**Cell Biology**	31	1.439
**Hematology**	31	1.439
**Pediatrics**	30	1.393
**Peripheral Vascular Diseases**	30	1.393
**Sport Sciences**	30	1.393
**Physiology**	25	1.161
**Respiratory System**	25	1.161
**Dentistry, Oral Surgery and Medicine**	24	1.114
**Radiology, Nuclear Medicine and Medical Imaging**	24	1.114
**Obstetrics and Gynecology**	23	1.068
**Gastroenterology and Hepatology**	22	1.021
**Health Care Sciences and Services**	21	0.975
**Parasitology**	21	0.975
**Urology and Nephrology**	21	0.975
**Ophthalmology**	20	0.929
**Biology**	17	0.789
**Biotechnology and Applied Microbiology**	17	0.789
**Environmental Sciences**	17	0.789
**Orthopedics**	17	0.789
**Rehabilitation**	17	0.789
**Chemistry, Multidisciplinary**	16	0.743
**Emergency Medicine**	16	0.743
**Engineering, Biomedical**	16	0.743
**Pathology**	16	0.743
**Tropical Medicine**	16	0.743
**Behavioral Sciences**	14	0.650
**Virology**	14	0.650
**Biochemical Research Methods**	13	0.604
**Critical Care Medicine**	13	0.604
**Geriatrics and Gerontology**	13	0.604
**Reproductive Biology**	13	0.604
**Food Science and Technology**	12	0.557
**Integrative and Complementary Medicine**	12	0.557
**Anesthesiology**	11	0.511
**Otorhinolaryngology**	11	0.511
**Rheumatology**	11	0.511
**Chemistry, Analytical**	10	0.464
**Developmental Biology**	10	0.464
**Plant Sciences**	10	0.464
**Psychology**	10	0.464
**Psychology, Clinical**	10	0.464
**Allergy**	9	0.418
**Medicine, Legal**	9	0.418
**Nursing**	9	0.418
**Veterinary Sciences**	9	0.418
**Biophysics**	8	0.371
**Health Policy and Services**	8	0.371
**Medical Laboratory Technology**	8	0.371
**Anatomy and Morphology**	7	0.325
**Chemistry, Organic**	7	0.325
**Mycology**	7	0.325
**Nanoscience and Nanotechnology**	7	0.325
**Computer Science, Interdisciplinary Applications**	6	0.279
**Psychology, Experimental**	6	0.279
**Chemistry, Applied**	5	0.232
**History and Philosophy Of Science**	5	0.232
**Mathematical and Computational Biology**	5	0.232
**Medical Informatics**	5	0.232
**Substance Abuse**	5	0.232
**Zoology**	5	0.232
**Microscopy**	4	0.186
**Neuroimaging**	4	0.186
**Nuclear Science and Technology**	4	0.186
**Psychology, Multidisciplinary**	4	0.186
**Transplantation**	4	0.186
**Andrology**	3	0.139
**Anthropology**	3	0.139
**Cell and Tissue Engineering**	3	0.139
**Computer Science, Artificial Intelligence**	3	0.139
**Computer Science, Information Systems**	3	0.139
**Criminology and Penology**	3	0.139
**Ethics**	3	0.139
**Evolutionary Biology**	3	0.139
**Multidisciplinary Sciences**	3	0.139
**Primary Health Care**	3	0.139
**Social Sciences, Biomedical**	3	0.139
**Acoustics**	2	0.093
**Audiology and Speech-Language Pathology**	2	0.093
**Ecology**	2	0.093
**Education, Scientific Disciplines**	2	0.093
**Ergonomics**	2	0.093
**Gerontology**	2	0.093
**Materials Science, Multidisciplinary**	2	0.093
**Psychology, Applied**	2	0.093
**Psychology, Biological**	2	0.093
**Psychology, Development**	2	0.093
**Social Work**	2	0.093
**Agriculture, Dairy and Animal Science**	1	0.046
**Agronomy**	1	0.046
**Biodiversity Conservation**	1	0.046
**Chemistry, Inorganic and Nuclear**	1	0.046
**Chemistry, Physical**	1	0.046
**Economics**	1	0.046
**Education and Educational Research**	1	0.046
**Engineering, Chemical**	1	0.046
**Engineering, Industrial**	1	0.046
**Entomology**	1	0.046
**Environmental Studies**	1	0.046
**Family Studies**	1	0.046
**Fisheries**	1	0.046
**Hospitality, Leisure, Sport and Tourism**	1	0.046
**Humanities, Multidisciplinary**	1	0.046
**Imaging Science and Photographic Technology**	1	0.046
**Information Science and Library Science**	1	0.046
**Management**	1	0.046
**Marine and Freshwater Biology**	1	0.046
**Materials Science, Biomaterials**	1	0.046
**Mathematics, Interdisciplinary Applications**	1	0.046
**Medical Ethics**	1	0.046
**Optics**	1	0.046
**Paleontology**	1	0.046
**Philosophy**	1	0.046
**Physics, Particles and Fields**	1	0.046
**Robotics**	1	0.046
**Social Issues**	1	0.046
**Social Sciences, Interdisciplinary**	1	0.046
**Social Sciences, Mathematical Methods**	1	0.046
**Statistics and Probability**	1	0.046
**Water Resources**	1	0.046
**Total**	2154	
**These journals are not indexed in WOS**	817	
**2016–2023**		
**WOS Categories**	Count	(Count/Total) × 100
**Pharmacology and Pharmacy**	159	6.257
**Medicine, General and Internal**	106	4.172
**Public, Environmental, and Occupational Health**	103	4.054
**Neurosciences**	99	3.896
**Oncology**	79	3.109
**Biochemistry and Molecular Biology**	65	2.558
**Clinical Neurology**	65	2.558
**Immunology**	63	2.479
**Microbiology**	62	2.440
**Toxicology**	61	2.401
**Infectious Diseases**	60	2.361
**Medicine, Research and Experimental**	57	2.243
**Psychiatry**	56	2.204
**Endocrinology and Metabolism**	48	1.889
**Chemistry, Medicinal**	46	1.810
**Pediatrics**	46	1.810
**Surgery**	46	1.810
**Cardiac and Cardiovascular System**	44	1.732
**Nutrition and Dietetics**	44	1.732
**Dermatology**	40	1.574
**Cell Biology**	39	1.535
**Genetics and Heredity**	39	1.535
**Respiratory System**	36	1.417
**Hematology**	34	1.338
**Sport Sciences**	34	1.338
**Health Care Sciences and Services**	32	1.259
**Urology and Nephrology**	30	1.181
**Gastroenterology and Hepatology**	28	1.102
**Physiology**	28	1.102
**Radiology, Nuclear Medicine and Medical Imaging**	27	1.063
**Obstetrics and Gynecology**	26	1.023
**Orthopedics**	26	1.023
**Engineering, Biomedical**	25	0.984
**Parasitology**	25	0.984
**Dentistry, Oral Surgery and Medicine**	24	0.945
**Biology**	23	0.905
**Peripheral Vascular Diseases**	23	0.905
**Environmental Sciences**	22	0.866
**Integrative and Complementary Medicine**	22	0.866
**Ophthalmology**	21	0.826
**Tropical Medicine**	20	0.787
**Pathology**	19	0.748
**Behavioral Sciences**	17	0.669
**Chemistry, Multidisciplinary**	17	0.669
**Food Science and Technology**	17	0.669
**Critical Care Medicine**	16	0.630
**Emergency Medicine**	16	0.630
**Psychology, Clinical**	16	0.630
**Allergy**	15	0.590
**Biotechnology and Applied Microbiology**	15	0.590
**History and Philosophy Of Science**	15	0.590
**Rehabilitation**	15	0.590
**Biochemical Research Methods**	14	0.551
**Chemistry, Analytical**	14	0.551
**Health Policy and Services**	13	0.512
**Otorhinolaryngology**	13	0.512
**Psychology, Experimental**	13	0.512
**Substance Abuse**	13	0.512
**Geriatrics and Gerontology**	12	0.472
**Medicine, Legal**	12	0.472
**Reproductive Biology**	12	0.472
**Psychology**	11	0.433
**Virology**	11	0.433
**Nanoscience and Nanotechnology**	10	0.394
**Plant Sciences**	10	0.394
**Veterinary Sciences**	10	0.394
**Anesthesiology**	9	0.354
**Mathematical and Computational Biology**	9	0.354
**Medical Laboratory Technology**	9	0.354
**Mycology**	9	0.354
**Nursing**	9	0.354
**Rheumatology**	9	0.354
**Biophysics**	8	0.315
**Computer Science, Artificial Intelligence**	8	0.315
**Medical Informatics**	8	0.315
**Multidisciplinary Sciences**	8	0.315
**Chemistry, Organic**	7	0.275
**Zoology**	7	0.275
**Anatomy and Morphology**	6	0.236
**Computer Science, Interdisciplinary Applications**	6	0.236
**Developmental Biology**	6	0.236
**Materials Science, Biomaterials**	6	0.236
**Transplantation**	6	0.236
**Anthropology**	5	0.197
**Chemistry, Applied**	5	0.197
**Criminology and Penology**	5	0.197
**Education, Scientific Disciplines**	5	0.197
**Microscopy**	5	0.197
**Neuroimaging**	5	0.197
**Nuclear Science and Technology**	5	0.197
**Psychology, Multidisciplinary**	5	0.197
**Andrology**	4	0.157
**Audiology and Speech-Language Pathology**	4	0.157
**Cell and Tissue Engineering**	4	0.157
**Evolutionary Biology**	4	0.157
**Psychology, Biological**	4	0.157
**Psychology, Development**	4	0.157
**Social Sciences, Biomedical**	4	0.157
**Ethics**	3	0.118
**Materials Science, Multidisciplinary**	3	0.118
**Medical Ethics**	3	0.118
**Primary Health Care**	3	0.118
**Psychology, Applied**	3	0.118
**Robotics**	3	0.118
**Acoustics**	2	0.079
**Computer Science, Information Systems**	2	0.079
**Ecology**	2	0.079
**Education, Special**	2	0.079
**Ergonomics**	2	0.079
**History**	2	0.079
**Psychology, Psychoanalysis**	2	0.079
**Agriculture, Dairy and Animal Science**	1	0.039
**Agronomy**	1	0.039
**Asian Studies**	1	0.039
**Biodiversity Conservation**	1	0.039
**Chemistry, Inorganic and Nuclear**	1	0.039
**Chemistry, Physical**	1	0.039
**Computer Science, Theory and Methods**	1	0.039
**Electrochemistry**	1	0.039
**Engineering, Industrial**	1	0.039
**Engineering, Multidisciplinary**	1	0.039
**Entomology**	1	0.039
**Environmental Studies**	1	0.039
**Fisheries**	1	0.039
**Green and Sustainable Science and Technology**	1	0.039
**Hospitality, Leisure, Sport and Tourism**	1	0.039
**Humanities, Multidisciplinary**	1	0.039
**Imaging Science and Photographic Technology**	1	0.039
**Law**	1	0.039
**Linguistics**	1	0.039
**Marine and Freshwater Biology**	1	0.039
**Mathematics, Interdisciplinary Applications**	1	0.039
**Optics**	1	0.039
**Paleontology**	1	0.039
**Physics, Applied**	1	0.039
**Physics, Particles and Fields**	1	0.039
**Psychology, Educational**	1	0.039
**Psychology, Social**	1	0.039
**Social Sciences, Interdisciplinary**	1	0.039
**Social Sciences, Mathematical Methods**	1	0.039
**Spectroscopy**	1	0.039
**Transportation Science and Technology**	1	0.039
**Water Resources**	1	0.039
**Total**	2541	
**These journals are not indexed in WOS**	888	
